# Phosphinoindenyl and phosphazidoindenyl complexes of lanthanum and samarium: synthesis, characterisation, and hydroamination catalysis†

**DOI:** 10.1039/d1dt03219d

**Published:** 2021-12-24

**Authors:** Matthias R. Steiner, Johann A. Hlina, Johanna M. Uher, Roland C. Fischer, Dmytro Neshchadin, Theresa Wilfling

**Affiliations:** Institute of Inorganic Chemistry, Graz University of Technology Stremayrgasse 9 8010 Graz Austria johann.hlina@tugraz.at; Institute of Physical and Theoretical Chemistry, Graz University of Technology Stremayrgasse 9 8010 Graz Austria

## Abstract

The phosphinoindenyl rare-earth metal complexes [1-(Ph_2_P)-η^5^-C_9_H_6_]_2_Ln^III^N(SiMe_3_)_2_, Ln = La (1-La), Sm (1-Sm), were prepared by heating two equivalents of 1-(Ph_2_P)C_9_H_7_ with Ln^III^[N(SiMe_3_)_2_]_3_ in toluene at 100 °C. The treatment of 1-La with one equivalent of benzonitrile gave (PhCN)[1-(Ph_2_P)-η^5^-C_9_H_6_]_2_La^III^N(SiMe_3_)_2_, 2, while no adduct was formed in case of the samarium derivative 1-Sm. The reaction of 1-La and 1-Sm with two equivalents of benzyl azide yielded the (phosphazido)indenyl complexes {1-[BnN_3_-κ*N*(Ph_2_)P]-η^5^-C_9_H_6_}{1-[BnN_3_-κ^2^*N*,*N*′(Ph_2_)P]C_9_H_6_}Ln^III^N(SiMe_3_)_2_, Ln = La (3-La), Sm (3-Sm), respectively. The five complexes catalyse the intramolecular hydroamination/cyclisation of 2,2-diphenylpent-4-ene-1-amine using 2% catalyst loading. All compounds were characterised by NMR and UV-Vis spectroscopy, single-crystal X-ray diffraction, and elemental analysis and DFT calculations were performed for 3-La.

## Abstract 2

We describe phosphino- and phosphazidoindenyl rare-earth metal complexes and their application in hydroamination catalysis.

## Graphical abstract

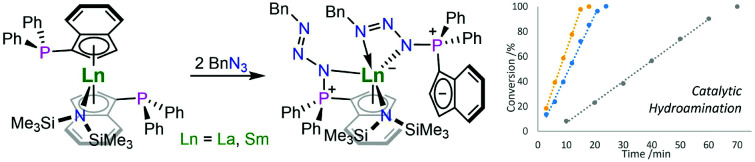

## Introduction

**Fig. 1:**
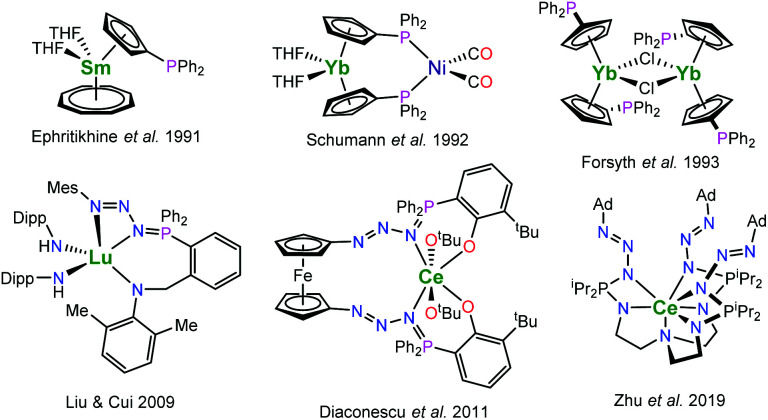
Selected examples of rare-earth metal (diphenylphosphino)cyclopentadienyl and phosphazide complexes (Dipp = C_6_H_3_-2,6-^i^Pr_2_, Mes = mesityl, Ad = adamantyl).^7,9,10,17–19^

Cyclopentadienyl ligands are among the work horses of organometallic rare-earth metal chemistry. But despite their prominence and the myriads of different cyclopentadienyl ligands, complexes featuring diorganophosphinyl-substituted cyclopentadienyl ligands remain comparably rare. Previously reported works include both monometallic complexes^1–8^ as well as heterometallic complexes (Fig. 1).^8–14^ In the latter, the phosphane moieties are employed as binding sites for late transition metals. Interestingly, these investigations focussed on the synthetically challenging diorganophosphinyl-substituted cyclopentadienyl ligands. However, the indenyl derivatives remained widely unexplored. In terms of diorganophosphorus-substituted indenyl complexes, examples of the rare earth complexes are limited to phosphazenes and phosphonium derivatives instead, in which the phosphorus is utilised to bind an additional donor to engage in chelation along with the indenyl group. As part of these works, rare-earth metal phosphazenoindenyl complexes have also been shown to be active hydroamination catalysts.^15^

Related to the phosphazenes are the phosphazides, the direct addition products of organic azides with tertiary phosphines. These key intermediates of the Staudinger reaction, which readily decompose to phosphazenes under dinitrogen release, may be stabilized by metal ions to which they act as a mono- or bidentate ligands.^16^ The number of rare-earth phosphazide complexes is very limited; selected examples are depicted in Fig. 1.^17–19^ Liu and Cui reported a lutetium complex featuring a phosphazidoamide ligand, [MesN_3_PPh_2_(*o*-C_6_H_4_)CH_2_N(CH_3_-2,6-Me_2_)]Lu^III^(NHDipp)_2_ (Mes = mesityl, Dipp = 2,6-diisopropylphenyl), which was prepared by treatment of the parent lutetium phosphinoamide complex with mesityl azide.^17^ Later, Diaconescu and co-workers demonstrated that use of a 1,1′-diazidoferrocene gives access to ferrocene-bridged phosphazidophenolate complexes of cerium such as Fc-1,1′-[N_3_PPh_2_(C_6_H_3_-3-^*t*^Bu-2-O)_2_Ce^IV^(O^*t*^Bu)_2_ (Fc = ferrocene).^18^ In this case it was shown that such complexes may be converted into the corresponding phosphazene complexes under release of dinitrogen at elevated temperatures. The consecutive loss of dinitrogen from a bis(phosphazene) ligand was recently reported for a uranium compound.^20^ In contrast to that, Zhu *et al.* reported the TREN-based phosphazidoamide cerium complex N(CH_2_CH_2_NP^i^Pr_2_N_3_Ad)_3_Ce^III^ (Ad = adamantyl), which exhibited significantly higher thermal stability.^19^ However, using trimethylsilyl azide instead yielded the corresponding phosphazene complex N(CH_2_CH_2_NP^i^Pr_2_NSiMe_3_)_3_Ce^III^ underlining that the stability of the phosphazides strongly depends on the nature of the substituent on the azide.

In this work, we present the first examples of rare-earth phosphinoindenyl and phosphazidoindenyl complexes and our studies exploring their reactivity including their activity to catalyse intramolecular hydroamination reactions.

## Results and discussion

### Synthesis

**Fig. 2:**
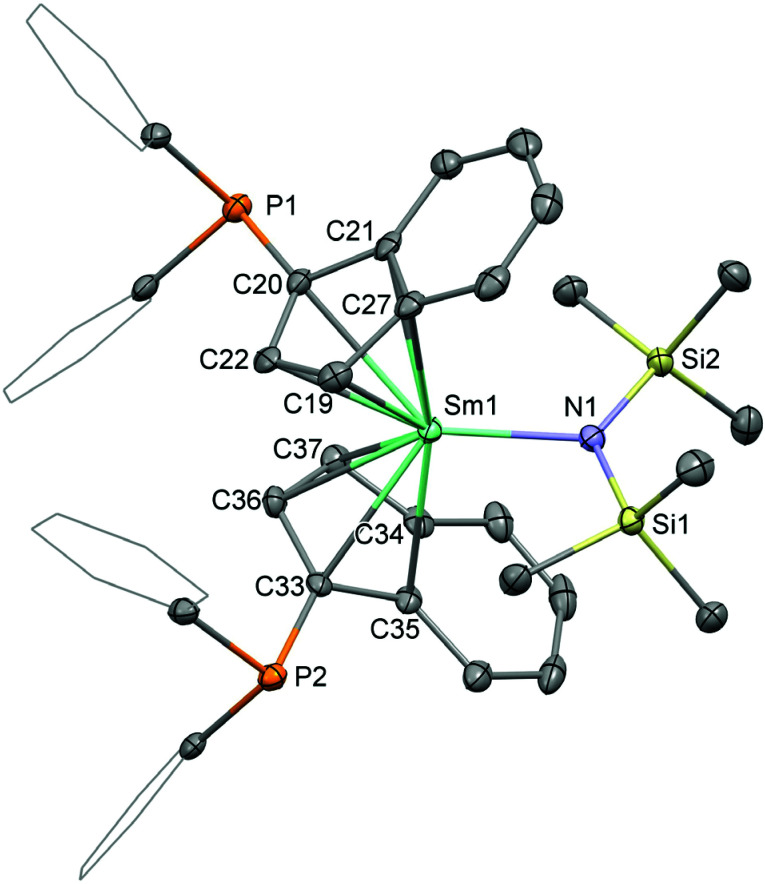
Molecular structure of 1-Sm. Hydrogen atoms are omitted and selected carbon atoms are depicted as wireframe for clarity. Thermal ellipsoids drawn at 50% probability. Selected distances (Å) and angles (deg): Sm1–N1: 2.239(3), Sm1–C19: 2.732(4), Sm1–C20: 2.759(3), Sm1–C21: 2.814(4), Sm1–C22: 2.751(3), Sm1–C27: 2.803(4), Sm1–C33: 2.740(3), Sm1–C34: 2.795(3), Sm1–C35: 2.816(3), Sm1–C36: 2.728(3), Sm1–C37: 2.698(3), C20–P1: 1.816(4), C33–P2: 1.815(4), N1–Sm1–ct1: 116.81, Sm1–N1–Si1: 112.8(2), Sm1–N1–Si2: 118.6(2).

**Scheme 1:**
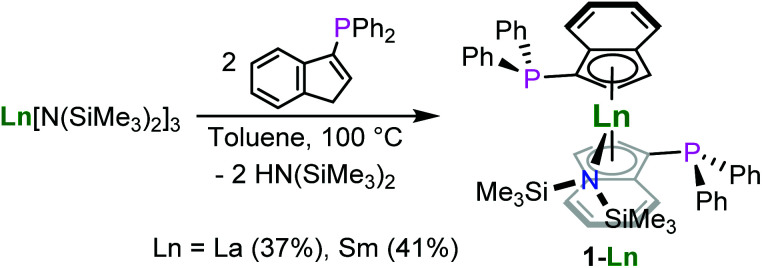
Preparation of the phosphinoindenyl lanthanum (1-La) and samarium complexes (1-Sm).

The synthesis of the bis(phosphinoindenyl) complexes [1-(Ph_2_P)-η^5^-C_9_H_6_]_2_Ln^III^N(SiMe_3_)_2_, Ln = La (1-La), Sm (1-Sm), was performed by heating solutions of 1-(Ph_2_P)C_9_H_7_ and Ln^III^[N(SiMe_3_)_2_]_3_ (Ln = La, Sm) in a 2 : 1 molar ratio in toluene at 100 °C for 18 h (Scheme 1). The compounds 1-La and 1-Sm were isolated as pale yellow and red solids after precipitation from diethyl ether in 37 and 41% yield, respectively. The molecular structures of the complexes 1-La and 1-Sm (Fig. 2), determined by single crystal X-ray diffraction, exhibited the *rac*-isomers. The NMR spectroscopic data also showed only a single set of ligand signals and no side differentiation for the trimethylsilyl groups of the hexamethyldisilazide for both the lanthanum and samarium compounds. So, we conclude that in solution at ambient temperature we observe the *rac*-isomers. The attempted synthesis of [1-(Ph_2_P)-η^5^-C_9_H_6_]_3_La^III^ using three equivalents of the phosphinoindene was unsuccessful. After 3d of heating to 100 °C 1-La was found to be the main product along with unidentified decomposition products including oxidised phosphane moieties as indicated by ^31^P NMR spectroscopy.

**Scheme 2:**
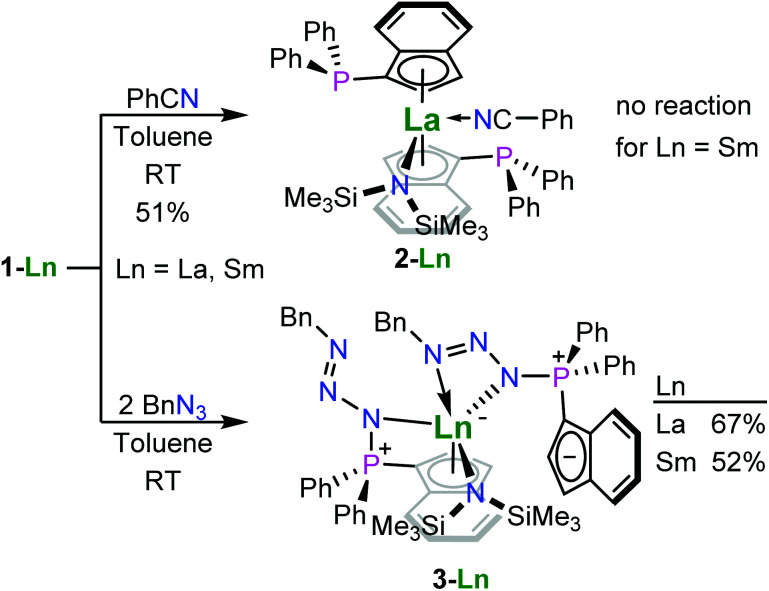
The syntheses of the benzonitrile adduct of the phosphinoindenyl lanthanum complex (2) and the phosphazidoindenyl lanthanum (3-La) and samarium (3-Sm) complexes.

In the context of hydroamination/cyclisation reactions, Marks and co-workers observed product inhibition for rare-earth metallocene complexes, which also resulted in a change of observed reaction orders from zero-order to apparent first-order rate kinetics.^21–25^ In order to explore such changes in reaction behaviour by limiting accessibility of the rare-earth metal centre, we used benzonitrile as additional ligand. This compound was chosen for typically binding *via* the nitrogen atom and presumably strong enough to remain coordinated under the conditions of hydroamination reactions. For this purpose we treated 1-La and 1-Sm with one equivalent of benzonitrile in benzene at ambient temperature yielding the benzonitrile adduct 2, [1-(Ph_2_P)-η^5^-C_9_H_6_]_2_La^III^N(SiMe_3_)_2_(NCPh), in case of the lanthanum derivative in 51% yield, while the samarium complex did not exhibit any coordination of benzonitrile (Scheme 2). We attribute this to the fact that the samarium(iii) ion is smaller than lanthanum(iii), so the present ligand sphere conceals the rare-earth metal ion more effectively in case of the smaller ion. The use of two equivalents of benzonitrile also yielded 2 without apparent coordination of the second benzonitrile equivalent.

The crystal structures of 1-La and 1-Sm exhibited non-coordinating phosphane donors (Fig. 2), which prompted us to explore the possibility towards post-complexation derivatization of the ligand system. Therefore, we treated 1-La and 1-Sm with two equivalents of benzyl azide at ambient temperature. Since the phosphanes were not involved in metal bonding and the metal centres are rather concealed by the ligand sphere present, we expected the reactions to proceed to the corresponding phosphazenes. However, no gas evolution was observed and crystallographic analysis later revealed that the reaction already stopped after forming the phosphazide complexes. Therefore, the reactions actually yielded the yellow phosphazide-substituted indenyl complexes {1-[BnN_3_-κ*N*-(Ph_2_)P]-η^5^-C_9_H_6_}{1-[BnN_3_-κ^2^*N*,*N*′-(Ph_2_)P]C_9_H_6_}Ln^III^N(SiMe_3_)_2_, Ln = La (3-La), Sm (3-Sm), in 67 and 52% yield, respectively (Scheme 2). The isolated compounds were found to decompose under inert conditions even when stored at −20 °C, with 3-Sm appearing to be less stable than 3-La forming significant amounts of hexamethyldisilazane within a week as the only identifiable decomposition product. The parent compounds did not exhibit any decomposition when stored under the same conditions, which is why we exclude mere hydrolysis. In both cases, the solid-state structure shows one of the two phosphazidoindenyl ligands coordinated *via* the cyclopentadienyl substructure and the phosphorus-bound nitrogen atom, while the second ligand is coordinated through two of the azide nitrogen atoms with the indenyl group not bound to the rare-earth metal centre. So, these complexes show formal intramolecular charge separation with the positive charge of the rare-earth metal centre being compensated by the tethered not coordinating indenyl anion. Rare-earth metal phosphazide complexes have previously been reported by the groups of Cui and Diaconescu and coordinate to the metal centres *via* one or two of the azide nitrogen atoms (see Fig. 1 for examples).^17–19^ However, there are no other phosphazide complexes of d- and f-block metals with cyclopentadienyl-functionalised triorganophosphorus groups to the best of our knowledge. Considering the examples of the groups of Diaconescu^18^ on cerium complexes and Hayes on uranium complexes,^20^ we attempted the thermal decomposition of the phosphazide complexes 3-La and 3-Sm to form the corresponding phosphazenes under release of dinitrogen. For this purpose, we heated solutions of the compounds in toluene to 70 °C. Decomposition was indicated by gas evolution, but yielded inseparable mixtures of products. In contrast to the previously reported examples, the complexes 3-La and 3-Sm feature a strongly basic hexamethyldisilazide group which may be involved in the formation of side-products.

### Crystallography

Crystal samples of the phosphinoindenyl complexes 1-La and 1-Sm suitable for single-crystal X-ray diffractometry were grown from concentrated solutions in toluene or benzene layered with pentane. Compound 1-La crystallised in the triclinic space group *P*1̄ (Fig. S1 in the ESI†) as colourless and 1-Sm (Fig. 2) in the monoclinic space group *P*2_1_/*n* as dark red blocks, respectively. Both structures exhibit *rac*-isomers but differ in the orientation of one of the diphenylphosphinyl groups, which is also disordered in 1-La. We also collected data exhibiting 1-La isostructural to 1-Sm, but unfortunately the data was of poor quality. The Ln–C bond distances of the rare-earth metals to the cyclopentadienyl substructure are in range of 2.822(5) to 2.920(9) Å in 1-La and 2.698(3) to 2.816(3) Å in 1-Sm and the corresponding distances of the metal centre to the calculated centroids, ct1 and ct2, are 2.5470(4) and 2.5738(3) Å for 1-La and 2.4755(5) and 2.4920(4) Å for 1-Sm, respectively. The hexamethyldisilazide group resides in between the arene sections of the indenyl ligands, with Ln–N bond distances of 2.339(3) Å (La1–N1, 1-La) and 2.239(3) Å (Sm1–N1, 1-Sm), resulting in ct1–Ln–ct2 bending angles of 119.22(1) (ct1–La1–ct2, 1-La) and 123.62(1) ° (ct1–Sm1–ct2, 1-Sm). The relative angles of the planes based on the five carbon atoms of the cyclopentadienyl substructures are 62.5(5)° in case of 1-La and 60.5(1) ° in that of 1-Sm. The bond distances of the phosphorus atoms to the indenyl carbon atoms are 1.83(2) (P1–C22) and 1.807(4) (P2–C1) in 1-La and 1.849(2) (P1–C20) and 1.834(4) (P2–C33) in 1-Sm and very similar to the corresponding bond distance in bis[1-(diphenylphosphino)indenyl]ferrocene with 1.829(3) Å.^26^

**Fig. 3:**
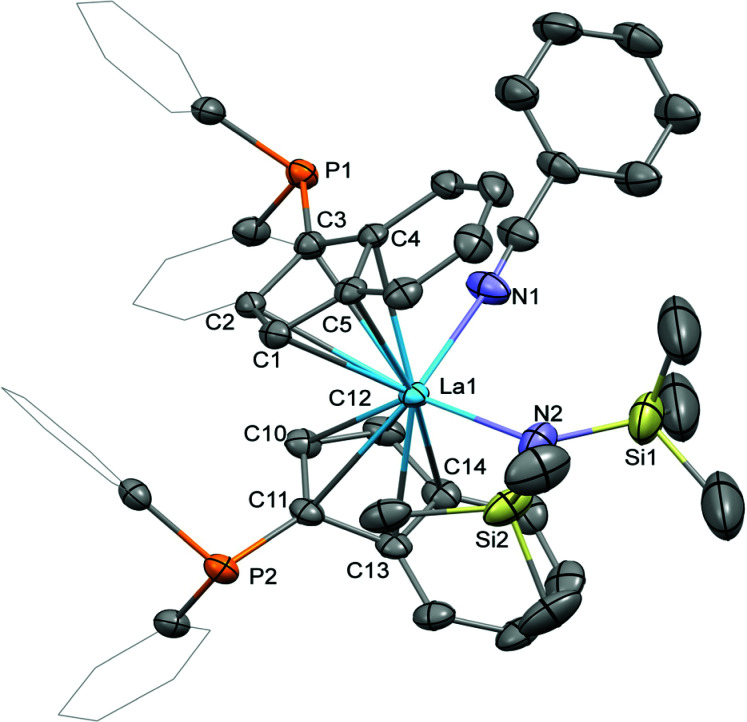
Molecular structure of 2. Hydrogen atoms are omitted and selected carbon atoms are depicted as wireframe for clarity. Thermal ellipsoids drawn at 50% probability. Selected distances (Å) and angles (deg): La1–N1: 2.619(3), La1–N2: 2.369(3), La1–C1: 2.863(3), La1–C2: 2.933(2), La1–C3: 2.938(2), La1–C4: 2.894(3), La1–C5: 2.863(3), La1–C10: 2.860(3), La1–C11: 2.849(3), La1–C12: 2.852(4), La1–C13: 2.909(3), La1–C14: 2.897(3), C3–P1: 1.809(3), C11–P2: 1.808(3), N1–La1–N2: 99.86(9), La1–N2–Si1: 128.2(2), La1–N2–Si2: 111.1(1).

Crystallisation of the benzonitrile adduct 2 from benzene/diethyl ether at ambient temperature yielded suitable material for crystallographic analysis (Fig. 3). The benzonitrile adduct 2 crystallised in the monoclinic space group *C*2/*c* in the form of pale yellow plates. The molecular structure exhibits the diphenylphosphinyl groups on opposite sides of the complex with the benzo substructures of the indenyl ligands on the same side. In comparison with 1-La, the La–C bond distances to the two indenyl ligands in 2, 2.850(3) to 2.938(2) Å, as well as the lanthanum-centroids distances, 2.606 (La1–ct1) and 2.634 Å (La1–ct2), are slightly elongated due to the coordination of the benzonitrile. Similarly, the La1–N2 bond distance to the hexamethyldisilazide is increased to 2.369(2) Å. The benzonitrile itself exhibits a La1–N1 bond distance of 2.611(3) Å with an N1–La1–N2 angle to the hexamethyldisilazide nitrogen atom of 99.92(9)°. With 122.6(2)° the ct1–La–ct2 angle is very similar to those in 1-La and 1-Sm and the angle between the two cyclopentadienyl planes is lower with 56.9(3)° than in both these complexes.

**Fig. 4:**
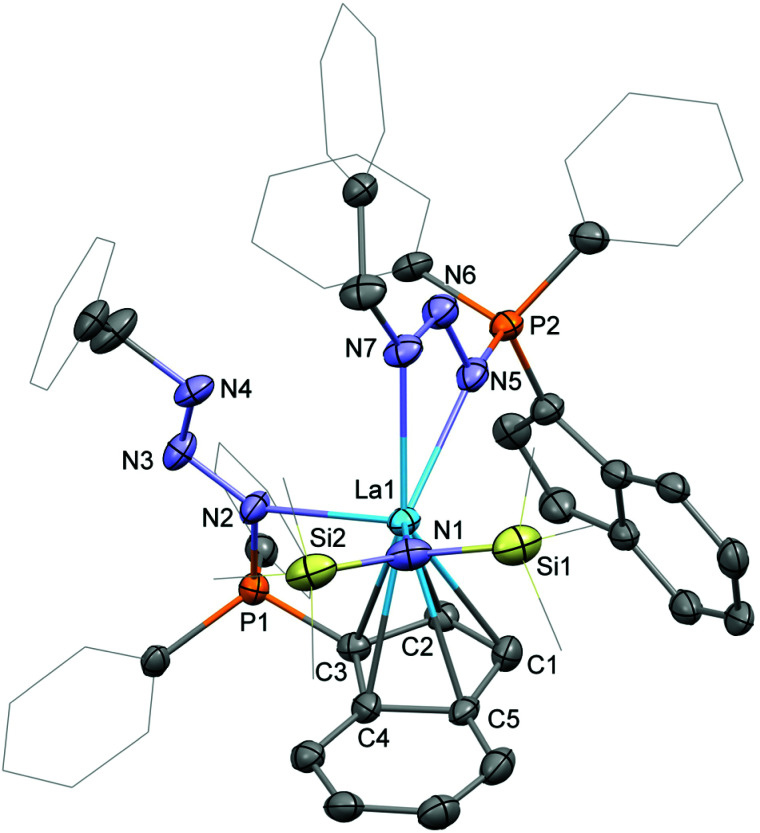
Molecular structure of 3-La. Hydrogen atoms are omitted and selected carbon atoms are depicted as wireframe for clarity. Thermal ellipsoids drawn at 50% probability. Selected distances (Å) and angles (deg): La1–N1: 2.342(4), La1–N2: 2.560(5), La1–N5: 2.615(5), La1–N7: 2.634(6), N2–N3: 1.389(8), N3–N4: 1.248(8), N5–N6: 1.383(8), N6–N7: 1.267(5), P1–N2: 1.641(6), P2–N5: 1.661(4), La1–C1: 2.962(7), La1–C2: 2.858(5), La1–C3: 2.843(6), La1–C4: 2.931(8), La1–C5: 2.996(7), C3–P1: 1.754(7), C31–P2: 1.723(7), N1–La1–N2: 122.6(2), N5–La1–N7: 48.9(2), N2–N3–N4: 110.9(5), N5–N6–N7: 110.1(5).

Crystals of the phosphazide complexes 3-La (Fig. 4) and 3-Sm (Fig. S2 in the ESI†) suitable for single-crystal X-ray diffraction were grown from toluene/diethyl ether at ambient temperature. Both complexes crystallise in form of yellow plates in the space group *P*1̄. As already mentioned above the two phosphazidoindenyl ligands bind in two different ways to the rare-earth metal centre. The first ligand is coordinated η^5^*via* the indenyl and the phosphorus-bound nitrogen atom. The second ligand coordinates through the two outer nitrogen atoms of the bent azide with the indenyl group not bound to the metal centre. The Ln–C bond distances for the coordinated indenyl moiety are in range of 2.843(6) to 2.996(7) Å in 3-La and 2.739(5) to 2.934(6) Å in 3-Sm and 2.6561(6) (La1–ct1) and 2.5606(4) (Sm1–ct1) Å for the corresponding centroids, respectively. This shows that in the phosphazide complexes the metal–ligand distances for the coordinated indenyl groups are longer than in 1-La, 2, or 1-Sm. Also, the hexamethyldisilazide groups exhibit slightly longer Ln–N bonds, 2.342(4) Å (La1–N1) in 3-La and 2.266(4) Å (Sm1–N1) in 3-Sm, in comparison to their parent complexes. A closer look at the coordinating azide nitrogen atoms shows the Ln–N bond distance for the ligand with the metal-bound indenyl at 2.560(5) Å (La1–N2) in 3-La and 2.496(4) Å (Sm1–N2) in 3-Sm. For the ligand with the non-coordinating indenyl group, the Ln–N distances are elongated to 2.615(5) (La1–N5) and 2.634(6) Å (La1–N7) in 3-La and 2.534(5) (Sm1–N5) and 2.523(5) Å (Sm1–N7) in 3-Sm.

Despite the different coordination modes of the two phosphazidoindenyl ligands, the structural differences in the PN_3_ sections are limited. The P–N bond distances are at 1.641(6) (P1–N2) and 1.661(4) Å (P2–N5) in 3-La and 1.638(4) Å (P1–N2) and 1.676(3) Å (P2–N5) in 3-Sm. Within the azide fragments, the phosphorus-bound nitrogen atoms exhibit single bonds to the central azide nitrogen atoms, 1.389(8) (N2–N3) and 1.383(8) Å (N5–N6) in 3-La and 1.397(6) (N2–N3) and 1.380(6) Å (N5–N6) in 3-Sm, which are followed by N=N double bonds connecting to the benzyl-substituted nitrogen atom, 1.248(8) (N3–N4) and 1.267(5) Å (N6–N7) in 3-La and 1.248(6) (N3–N4) and 1.273(4) Å (N6–N7) in 3-Sm. This is similar to the distances observed for the phosphazide moieties in the lutetium complex [MesN_3_PPh_2_(*o*-C_6_H_4_)CH_2_N(CH_3_-2,6-Me_2_)Lu^III^(NHDipp)_2_] by Liu and Cui as well as in the cerium compound Fc-1,1′-[N_3_PPh_2_(C_6_H_3_-3-^*t*^Bu-2-O)_2_Ce^IV^(O^*t*^Bu)_2_ by Diaconescu and co-workers.^17,18^

### NMR spectroscopy

**Fig. 5:**
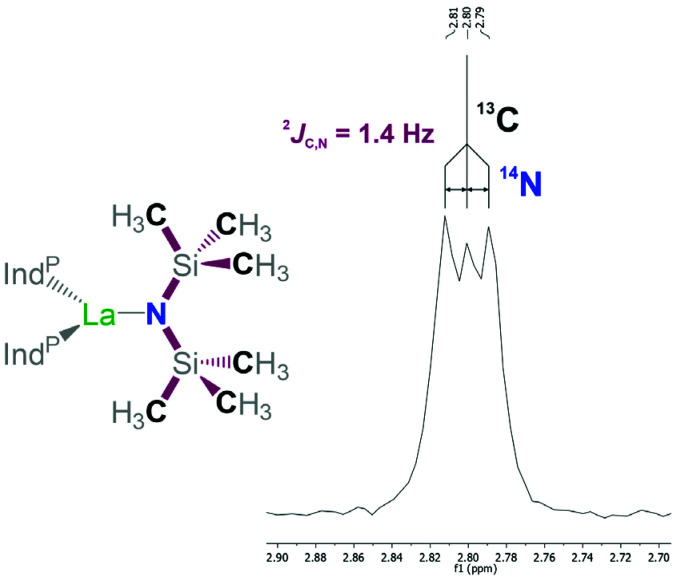
Detail view of the ^13^C{^1^H} NMR resonance assigned to the methyl groups in hexamethyldisilazide of 1-La in the corresponding NMR spectrum along with the substructure to illustrate the coupling between ^13^C and ^14^N. Ind^P−^ = 1-(Ph_2_P)C_9_H_6_^−^.

The ^1^H, ^13^C, and ^31^P NMR data indicates the presence of only the *rac*-isomers of 1-La and 1-Sm in solution at ambient temperature as solely a single resonance is observed for the two trimethylsilyl groups on the hexamethyldisilazide ligand along with only one set of signals for the 1-(diphenylphosphano)indenyl ligands. The ^1^H NMR resonances of 1-Sm experience paramagnetic shifting with the two protons on the cyclopentadienyl substructure of the indenyl being observed at 8.74 and 8.88 ppm. The remaining aromatic signals are less affected by the paramagnetic nature of the metal centre and found in range of 6.11 to 7.68 ppm. The resonance corresponding to the protons on the hexamethyldisilazide are observed at −5.75 ppm, while for 1-La the signal is observed at 0.25 ppm. The ^13^C{^1^H} NMR spectrum of 1-La exhibits the methyl resonance relating to the hexamethyldisilazide group at 2.8 ppm and appears to feature a faint coupling of 1.4 Hz to a nucleus with a spin of 1 resulting in a 1 : 1 : 1 triplet (Fig. 5). We attribute this to a coupling of ^13^C to ^14^N *via* two bonds. Due to the quadrupolar nature of the ^14^N nucleus its couplings are rarely encountered and typically require high symmetry or highly restricted movability to be observed. In the present case, we attribute this unusual observation to the limited possibilities of the hexamethyldisilazide group to move and rotate. In the context of rare-earth hexamethyldisilazide complexes a coupling to ^14^N was observed from a different perspective when ^171^Yb NMR data was recorded of [(Me_3_Si)_2_N]_2_Yb^II^(OEt_2_)_2_ at a magnitude of ^1^*J*_Yb,N_ = 117.6 Hz.^27^ Unfortunately, the corresponding ^13^C NMR data was not reported as part of this work. The observation of this coupling supports the hypothesis that changes and rearrangement in the coordination sphere around the lanthanum centre is comparably slow on an NMR time scale. In the case of 1-Sm the methyl resonance of the hexamethyldisilazide group was observed as a singlet at −4.7 ppm without any recognisable coupling. The ^31^P NMR measurements showed only a single resonance for both 1-La and 1-Sm at −26.0 and −20.1 ppm, respectively. The benzonitrile adduct 2 exhibits a situation similar to that in 1-La. This appears somewhat surprising since the coordination of a single benzonitrile molecule to 1-La would suggest an observable symmetry break. Considering that the solid state structure shows that the presence of the nitrile increases the bond distances of the other ligands, slightly widening up the coordination sphere and that the small nitrile group keeps the phenyl substituent at a distance to the indenyl ligands, it appears feasible that the nitrile may move comparably easy from the one side of the complex to the other side around its “back side”, opposite to the amide, without the need for significant rearrangement of the remaining ligands. In case of complex 2 the ^31^P NMR resonances was observed at −24.6 ppm.

**Fig. 6:**
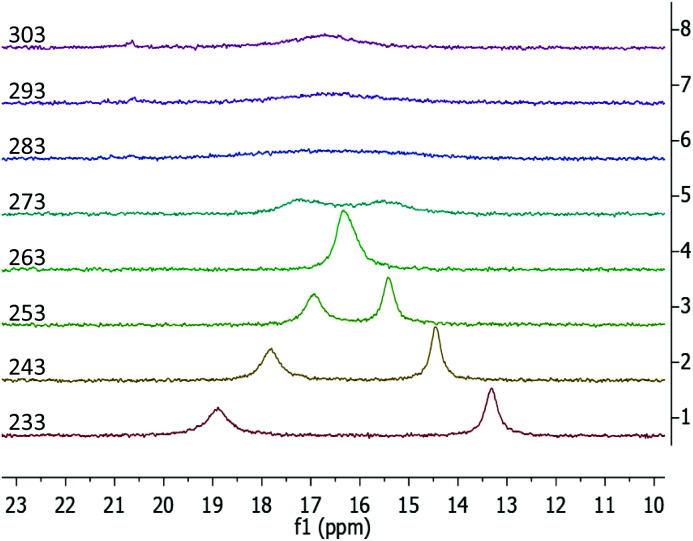
^31^P{^1^H} VT NMR spectra of 3-Sm recorded from a solution in toluene-*d*8 from 233 to 303 K in 10 K intervals.

The ^1^H NMR data of the phosphazide compound 3-La shows a single set of ligand signals. The protons of the trimethylsilyl groups are observed as a single resonance at −0.01 ppm. The methylene protons on the two benzyl groups appear as a single signal at 4.87 ppm, while the protons of the indenyl moiety give broad signals at 5.61 and 6.00 ppm respectively. We attribute this to a rapid exchange between coordination modes of the phosphazidoindenyl ligands. In case of the samarium derivative 3-Sm, we encounter a sharp, paramagnentically-shifted resonance for the hexamethyldisilazide protons at −2.65 ppm. Except for one broadened signal at 10.17 ppm, the remaining resonances overlap due to strong broadening, which renders separate integration impossible. Lowering the temperature of sample during the measurement underlines that the broadening of the signals results from dynamic behaviour, (see Fig. S28 and S33 in the ESI†). In terms of ^13^C NMR spectroscopy, even after prolonged measurement times no resonances could be observed for 3-Sm. The ^31^P NMR resonances of 3-La and 3-Sm are virtually the same and observed at 16.5 and 16.6 ppm, respectively, at ambient temperature. Both signals are strongly broadened with peak half widths of 185 Hz for the lanthanum and 383 Hz for the samarium derivative. The ^31^P variable temperature (VT) NMR spectroscopy for 3-La and 3-Sm showed coalescence temperatures at around 273 and 283 K, respectively. While the ^31^P VT NMR data of 3-La shows typical coalescence behaviour (see Fig. S29 in the ESI†), the samarium derivative 3-Sm appears to feature overlapping of the two ^31^P NMR signals below the coalescence temperature at 263 K (Fig. 6). We attribute this to the paramagnetic nature of the samarium(iii) ion, which may affect the two different phosphorus atoms differently when one of the attached indenyl moieties is coordinated to the samarium ion or while the other is not.

### Computational studies

**Fig. 7:**
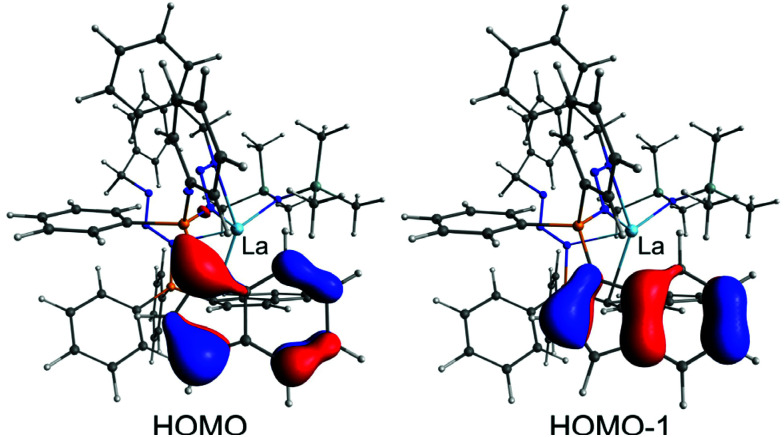
HOMO and HOMO−1 calculated for 3-La. The DFT calculations performed on the def2-TZVP level of theory with the M06-L functional. The isosurfaces are depicted at a value of 0.055.

In order to elucidate the bonding situation within the phosphazidoindenyl ligand with the non-coordinated indenyl group in the compounds 3-La and 3-Sm, we performed computational studies on the DFT level. The molecular structure of 3-La was used as a basis for the DFT calculations. The two highest occupied molecular orbitals, HOMO and HOMO−1, are located on the non-coordinated indenyl group (Fig. 7). The molecular orbitals consist of the two highest occupied orbitals of an indenyl anion, which feature two nodal planes perpendicular to the plane formed by the indenyl carbon atoms. This indicates electron delocalisation across the indenyl group and that it is rather an indenyl anion bound to the phosphorus atom through a single bond than a 1-indenylidene substituent connected to the phosphorus atom with a double bond. Moreover, the HOMO−2 and HOMO−6, which are the corresponding molecular orbitals of the coordinated indenyl group, are very similar (see Fig. S36 in the ESI†). The natural bond orbital (NBO) analysis revealed that the phosphorus atoms only bind through single bonds to the neighbouring atoms. Thus, they may best be described as phosphonium moieties. This supports the interpretation of the bonding situation in the non-coordinated indenyl group as anion. The phosphorus-bound nitrogen atoms, N2 and N5 in the molecular structure (Fig. 4), exhibit single bonds to their neighbouring nitrogen atoms, N3 and N6, respectively, and bear the second formal negative charge in each ligand. The N3–N4 and N6–N7 bonds within the azides exhibit double bond character, which agrees with the initial conclusion based in the N–N bond distances in the molecular structure.

### Hydroamination catalysis

**Scheme 3:**
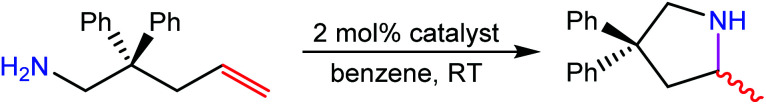
Catalytic hydroamination/cyclisation reaction of 2,2-diphenylpent-4-ne-1-amine using the complexes 1-La, 1-Sm, 2, 3-La, or 3-Sm as catalysts.

**Fig. 8:**
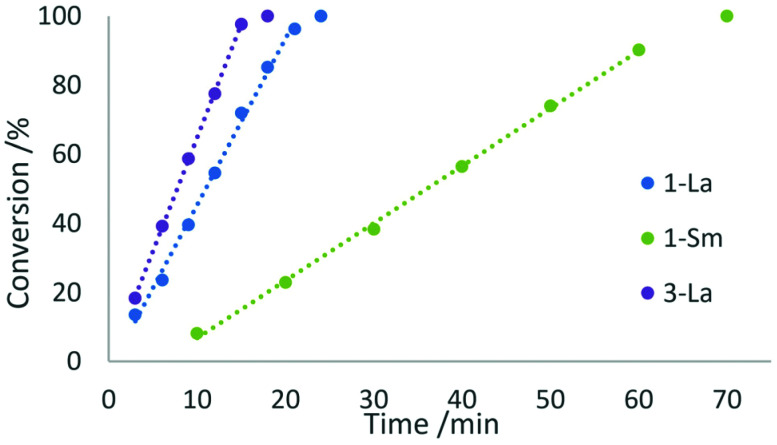
Conversion rates of the hydroamination/cyclisation of 2,2-diphenylpent-4-ene-1-amine using 2 mol% of 1-La, 1-Sm, or 3-La, as catalyst at ambient temperature in benzene. Trend lines are drawn as dotted lines and exclude the full conversion data point.

We investigated the activity of the five complexes in the intramolecular hydroamination/cyclisation reactions of 2,2-diphenylpent-4-ene-1-amine (Scheme 3). All complexes showed catalytic activity at ambient temperature using a catalyst loading of 2 mol% but exhibited certain differences in their reactivity. The complexes 1-La and 1-Sm showed full conversion after 24 min and 70 min which corresponds to turnover frequencies of 125 and 43 h^−1^, respectively. In both cases, the catalytic reaction follows a zero-order kinetic, which has previously been reported for other lanthanocene catalysts (Fig. 8).^25^

In contrast to this, the benzonitrile adduct 2 appears to rather follow a first-order rate kinetic at a turnover frequency of 20 h^−1^ with the conversion plateauing at 88% after 40 h (Fig. 8). A graph with ln(*c*_0_/*c*) (*c*_0_ = starting concentration, *c* = concentration at the time of measurement) *versus* time (Fig. S5 in the ESI†) supports a first-order kinetic for up to around 100 min of the reaction time after which the reaction rate decreases. The difference in reaction behaviour compared to the parent complex 1-La can be attributed to the strongly binding benzonitrile, which limits the accessibility of lanthanum.^25^

**Fig. 9:**
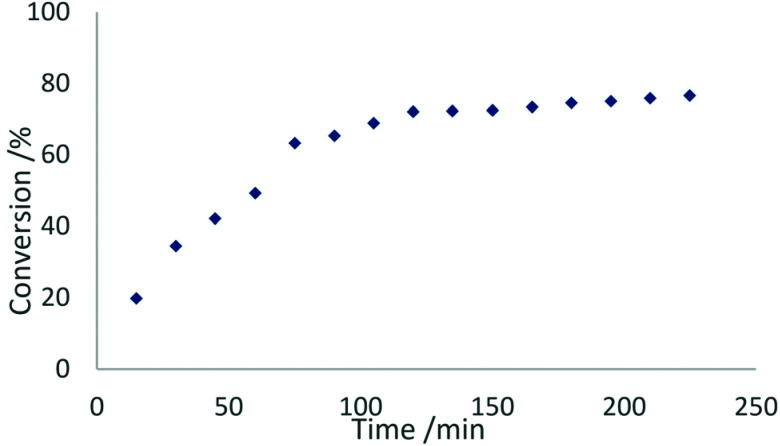
Conversion rates of the hydroamination/cyclisation of 2,2-diphenylpent-4-ene-1-amine using 2 mol% of 2 as catalyst at ambient temperature in benzene.

The phosphazide complex 3-La exhibited catalytic behaviour similar to that of 1-La, but with a slightly higher turnover frequency of 167 h^−1^ and full conversion observed after 18 minutes (Fig. 9). We presume that the higher reactivity of 3-La is related to the better accessibility of the metal centre, when compared to 1-La. In contrast to the lanthanum derivative, the samarium complex 3-Sm shows only very slow conversion with a turnover frequency of 5 h^−1^ in the hydroamination/cyclisation of 2,2-diphenylpent-4-ene-1-amine. The reaction was found to reach full conversion after around 11 h. Although smaller rare-earth metal ions commonly show slower conversion than larger rare-earth metal ions using the same ligand sphere, this difference in catalytic activity is surprising when comparing it with the activity of 1-La and 1-Sm. So the more pronounced difference in catalytic activity may originate in the changes of the ligand system itself rather than being a result of decomposition of the complex during catalysis.

## Conclusions

We demonstrated the preparation of the (diphenyl-phosphino)indenyl lanthanum (1-La) and samarium (1-Sm) complexes by protonolysis reactions from 3-(diphenylphos-phino)indene and Ln[N(SiMe_3_)_2_]_3_ (Ln = La, Sm). The two complexes were isolated as the *rac*-isomers. The ^13^C NMR analysis of 1-La also revealed a rarely observable ^14^N–^13^C coupling of the methyl-carbon atoms to the amide-nitrogen within the hexamethyldisilazide group. The lanthanum derivative 1-La readily adds benzonitrile to form 2, while 1-Sm appears to not coordinate benzonitrile. In order to explore post-complexation ligand derivatisation, 1-La and 1-Sm were treated with two equivalents of benzyl azide yielding the corresponding phosphazidoindenyl complexes 3-La and 3-Sm, respectively. To the best of our knowledge these are the first examples of complexes featuring phosphazidocyclopentadienyl-type ligands. In the solid state, these two complexes exhibit the two phosphazidoindenyl ligands coordinated in two different modes: with the indenyl and one phosphazide nitrogen bound to the rare-earth metal centre or the two phosphazide nitrogen atoms bound to the metal centre and the indenyl not coordinated. The ligands appear to change between the coordination modes in solution, which was further investigated using VT NMR spectroscopy. Both 3-La and 3-Sm decompose even under inert conditions at low temperature with the samarium derivative appearing less stable than the lanthanum derivative. The bonding situation within the phosphazido-indenyl ligands was investigated using DFT calculations along with an NBO analysis of 3-La and support an interpretation as phosphonium moiety bound to an indenyl anion and a negatively charged nitrogen atom.

We also investigated the reactivity of all the presented complexes towards the catalytic hydroamination/cyclisation of 2,2-diphenylpent-4-ene-1-amine. All complexes exhibited catalytic activity using 2 mol% catalyst loading at ambient temperature with the lanthanum derivatives being more active than their corresponding samarium congeners. The compounds 1-La, 1-Sm, 3-La, and 3-Sm showed zero-order reaction kinetics with turnover frequencies of up to 167 h^−1^ (3-La) while the benzonitrile adduct 2 exhibited first-order kinetics.

In summary, we introduced phosphinoindenyl ligands to rare-earth metal chemistry, demonstrated their utility towards ligand modification after complexation and showed that such complexes are active hydroamination catalysts.

## Experimental

### General details

All manipulations were carried out under an atmosphere of dry, oxygen-free nitrogen using standard Schlenk and glove box techniques. Deuterated benzene and toluene were dried by distillation from potassium under dry nitrogen. Dichloromethane and THF were purified by distillation from calcium hydride under nitrogen. All other solvents were purified by passing through columns of activated alumina.^28^ Other chemicals were obtained from different suppliers and used without further purification. Benzyl azide,^29^ Ln[N(SiMe_3_)_2_]_3_ (Ln = La, Sm),^30^ and 2,2-dimethylpent-4-ene-1-amine^31^ were prepared according to published procedures.

NMR spectra were recorded on a Varian INOVA 500, a Bruker AVANCE III 300, a Bruker AVANCE DPX 200 (for VT NMR spectroscopy), or an Rs2d 300 spectrometer and are referenced to Me_4_Si (^1^H, ^13^C), and 85% H_3_PO_4_ (^31^P). A numbering scheme for assignment of the signals relating to the indenyl ligands can be found in the ESI.† For X-ray structure analyses the crystals were mounted onto the tips of glass fibres. Data collection was performed with a Bruker-AXS SMART APEX CCD diffractometer using graphite-monochromated Mo-K_α_ radiation (0.71073 Å) or a Bruker APEX II diffractometer using a Incoatec microfocus sealed tube of Mo-K_α_ radiation (0.71073 Å) with a CCD area detector. The data were reduced to *F*_o_^2^ and corrected for absorption effects with SAINT^32^ and SADABS^33,34^ respectively. The structures were solved by direct methods and refined by full-matrix least-squares method (SHELXL97 or SHELXL19).^35^ If not noted otherwise all non-hydrogen atoms were refined with anisotropic displacement parameters. All hydrogen atoms were located in calculated positions to correspond to standard bond lengths and angles. Crystallographic data for the structures reported in this paper have been deposited with the Cambridge Crystallographic Data Centre as supplementary publication no. CCDC 2108459 (1-La), 2108458 (1-Sm), 2108460 (2), 2109013 (3-La), and 2110760 (3-Sm).† UV-Vis spectra were recorded on an Agilent Cary 60 UV-Vis spectrophotometer. Elementary analysis was carried out using a Heraeus VARIO ELEMENTAR. Computational studies were performed with the ORCA 4.0.1 and 4.2.1 program suits. The molecular structure derived from single-crystal X-ray diffraction data was used as basis for geometry optimisation and the calculations performed at the def2-TZVP level of theory for all atoms with the M06-L functional. The natural bond orbital analysis was performed using the NBO 7.0 software suit through ORCA.^36–38^

#### 3-(Diphenylphosphano)indene (HInd^P^)

The compound was prepared in adaptation of a published procedure.^39^ A Schlenk flask equipped with a stirrer bar was charged with indene (11.6 g, 100 mmol) and diethyl ether (100 ml) and cooled to −60 °C. Then n-butyllithium solution (44 ml, 110 mmol, 2.5 M in hexanes) was added to the stirred solution. The cooling bath was removed after the addition and the yellow solution allowed to warm to ambient temperature and stirred for 1 h. After that, the indenyllithium solution was added dropwise to a solution of chlorodiphenylphosphine (24.3 g, 110 mmol) in diethyl ether (40 ml) at −40 °C forming a white precipitate. The mixture was stirred for 1 h at the same temperature after the addition was finished and then allowed to warm to ambient temperature over the next 18 h. Then all volatiles were evaporated under reduced pressure and the yellow residue extracted with toluene. The yellow extract was concentrated under reduced pressure to a volume of *ca.* 40 ml followed by precipitation of the product by addition of pentane (60 ml). The supernatant yellow solution was filtered off and the remaining off-white solid washed three times with pentane. After drying under vacuum 19.6 g (65%) of HInd^P^ was isolated as an off-white powder. Analytical data was in accordance with published values.^39^

#### Bis[1-(diphenylphosphano)indenyl]bis(trimethylsilyl)amide lanthanum 1-La

A vial was charged with La[N(SiMe_3_)_2_]_3_ (2.48 g, 4.00 mmol), 3-(diphenylphosphino)indene (2.40 g, 8.00 mmol), and toluene (10 ml) and heated in a glove box to 100 °C for 18 h. After cooling to ambient temperature, all volatiles were evaporated under reduced pressure. The yellow residue was taken up in diethyl ether and pentane forming a precipitate, which was separated and washed with pentane. The precipitate was dried under vacuum yielding 1-La as a pale yellow microcrystalline solid (1.32 g, 37%). mp 208 °C. ^1^H NMR (*δ* in ppm, benzene-*d*6, 298 K): 0.25 (s, 18H, C*H*_3_), 6.21 (d, 2H, ^3^*J*_H,H_ = 3.4 Hz, H2), 6.49 (d, 2H, ^3^*J*_H,H_ = 3.4 Hz, H3), 7.10–7.16 (m, 8H, Ar–H), 7.24 (t, 2 H, ^3^*J*_H,H_ = 7.5 Hz. Ar–H), 7.30–7.35 (m, 4H, Ar–H), 7.38–7.46 (m, 8H, Ar–H), 7.75 (d, 2H, ^3^*J*_H,H_ = 8.3 Hz, Ar–H), 7.84 (d, 2H, ^3^*J*_H,H_ = 8.3 Hz, Ar–H), 8.06 (t, 4H, ^3^*J*_H,H_ = 7.5 Hz, Ar–H). ^13^C{^1^H} NMR (*δ* in ppm, benzene-*d*6, 298 K): 2.8 (t, ^2^*J*_N,C_ = 1.4 Hz, Si*C*H_3_), 105.4 (s, C9), 105.9 (d, ^2^*J*_P,C_ = 8.1 Hz, C2), 122.5 (d, ^1^*J*_P,C_ = 7.3 Hz, C1), 122.7 (s, C5), 123.4 (d, ^2^*J*_P,C_ = 25.4 Hz, C8), 127.9 (s, C6), 128.4 (s, C3), 128.6 (d, ^3^*J*_P,C_ = 6.7 Hz, C_m-Ph_), 129.1 (d, ^3^*J*_P,C_ = 8.8 Hz, C_m-Ph_), 129.4 (s, C_p-Ph_), 129.9 (s, C_p-Ph_), 132.3 (d, ^3^*J*_P,C_ = 18.8 Hz, C4), 134.1 (d, ^3^*J*_P,C_ = 4.4 Hz, C7), 135.0 (d, ^2^*J*_P,C_ = 22.5 Hz, C_o-Ph_), 136.2 (d, ^2^*J*_P,C_ = 22.2 Hz, C_o-Ph_), 138.2 (d, ^1^*J*_P,C_ = 6.7 Hz, C_i-Ph_), 140.3 (d, ^1^*J*_P,C_ = 14.8 Hz, C_i-Ph_) ^31^P{^1^H} NMR (*δ* in ppm, benzene-*d*6, 298 K): −26.0. UV-Vis: *λ*_max,1_ 315 nm (*ε*_1_ = 8.9 × 10^3^ L mol^−1^ cm^−1^, shoulder). Analysis calcd for C_48_H_50_LaNP_2_Si_2_ [897.96] C 64.20, H 5.61, N 1.56. Found: C 64.13, H 5.57, N 1.54.

#### Bis[1-(diphenylphosphanyl)indenyl]bis(trimethylsilyl)amide samarium 1-Sm

The same procedure was used as for 1-La employing Sm[N(SiMe_3_)_2_]_3_ (6.32 g, 10.0 mmol) and 3-(diphenylphosphano)indene (6.01 g, 20.0 mmol). The compound was isolated as a red microcrystalline solid (3.71 g, 41%). mp 195 °C. ^1^H NMR (*δ* in ppm, benzene-*d*6, 298 K): −5.75 (s, 18H, C*H*_3_), 6.20 (d, 2H, ^3^*J*_H,H_ = 8.1 Hz, Ar–H), 6.64 (d, 2H, ^3^*J*_H,H_ = 7.2 Hz, Ar–H), 6.96–7.08 (m, 6H, Ar–H), 7.18–7.33 (m, 6H, Ar–H), 7.42 (t, 2H, ^3^*J*_H,H_ = 7.5 Hz, Ar–H), 7.48–7.64 (m, 6H, Ar–H + H3), 8.74 (t, 2H, ^3^*J*_H,H_ = 7.5 Hz, Ar–H), 8.88 (t, 2H, ^3^*J*_H,H_ = 7.5 Hz, Ar–H), 20.50 (s, 2H, H2). ^13^C{^1^H} NMR (*δ* in ppm, benzene-*d*6, 298 K): −4.7 (s, Si*C*H_3_), 88.2 (s, C2), 91.1 (s, C3), 121.6 (s, C_Ar_), 122.1 (s, C_Ar_), 125.4 (s, C_Ar_), 128.6 (s, C_Ar_), 129.2 (s, C_Ar_), 129.6 (s, C_Ar_, 130.2 (s, C_Ar_), 130.5 (s, C_Ar_), 132.1 (s, C_Ar_), 134.2 (s, C_Ar_), 134.5 (s, C_Ar_), 135.5 (s, C_Ar_), 136.0 (s, C_Ar_), 136.9 (s, C_Ar_), 138.2 (s, C_Ar_). ^31^P{^1^H} NMR (*δ* in ppm, benzene-*d*6, 298 K): −20.1. UV-Vis: *λ*_max,1_ = 335 nm (*ε*_1_ = 1.2 × 10^4^ L mol^−1^ cm^−1^, shoulder), *λ*_max,2_ = 474 nm (*ε*_2_ = 5.9 × 10^2^ L mol^−1^ cm^−1^, broad band). Analysis calcd for C_48_H_50_NP_2_Si_2_Sm [909.41] C 63.40, H 5.54, N 1.54. Found: C 63.58, H 5.25, N 1.44.

#### Bis[1-(diphenylphosphanyl)indenyl]bis(trimethylsilyl)amide lanthanum benzonitrile adduct 2

A vial equipped with a stirrer bar was charged with 1-La (250 mg, 0.278 mmol) and benzene (5 ml) and a solution of benzonitrile (29 mg, 0.29 mmol) in benzene (5 ml) was added at ambient temperature under stirring. After stirring the solution for 18 h, all volatiles were evaporated under reduced pressure. The off-white residue was then crystallized from a concentrated solution in toluene layered with diethyl ether yielding 2 (142 mg, 51%) as pale yellow plates. mp 154 °C. ^1^H NMR (*δ* in ppm, benzene-*d*6, 298 K): 0.25 (s, 18H, C*H*_3_), 6.04 (dd, 2H, ^3^*J*_H,H_ = 3.2, 1.0 Hz, H2), 6.41 (d, 2H, ^3^*J*_H,H_ = 3.2 Hz, H3), 6.88 (t, 2H, ^3^*J*_H,H_ = 7.8 Hz, Ar–H), 6.96–7.01 (m, 6H, Ar–H), 7.02–7.06 (m, 3H, Ar–H), 7.10–7.15 (m, 5H, Ar–H), 7.23–7.27 (m, 2H,Ar–H), 7.28–7.34 (m, 5H, Ar–H), 7.58 (d, 2H, ^3^*J*_H,H_ = 7.5 Hz, Ar–H), 7.75 (d, 2H, ^3^*J*_H,H_ = 8.2 Hz, Ar–H), 7.80 (d, 2H, ^3^*J*_H,H_ = 8.2 Hz, Ar–H), 7.93 (t, 4H, ^3^*J*_H,H_ = 7.6 Hz, Ar–H). ^13^C{^1^H} NMR (*δ* in ppm, benzene-*d*6, 298 K): 4.4 (s, Si*C*H_3_), 104.5 (d, ^2^*J*_P,C_ = 8.6 Hz, C2), 105.1 (s, C9), 111.4 (C_Ar,CN-Ph_), 122.2 (d, ^3^*J*_P,C_ = 7.2 Hz, C1), 122.8 (s, C5), 123.2 (d, ^1^*J*_P,C_ = 26.1 Hz, C8), 125.7 (s, C_Ar,CN-Ph_), 128.4 (s, C3), 128.5 (d, ^1^*J*_P,C_ = 6.1 Hz, C_m-Ph_), 128.6 (s, C_Ar,CN-Ph_), 129.0 (d, ^1^*J*_P,C_ = 8.4 Hz, C_m-Ph_), 129.3 (s, C_p-Ph_), 129.7 (s, C_p-Ph_), 132.2 (d, ^3^*J*_P,C_ = 18.5 Hz, C4), 132.8 (C_Ar,CN-Ph_), 133.0 (d, ^3^*J*_P,C_ = 4.6 Hz, C7), 133.6 (C_Ar,CN-Ph_), 134.7 (d, ^2^*J*_P,C_ = 22.7 Hz, C_o-Ph_), 136.3 (d, ^2^*J*_P,C_ = 22.3 Hz, C_o-Ph_), 138.4 (d, ^4^*J*_P,C_ = 7.4 Hz, C_i-Ph_), 141.0 (d, ^2^*J*_P,C_ = 14.3 Hz, C_i-Ph_). ^31^P{^1^H} NMR (*δ* in ppm, benzene-*d*6, 298 K): −24.6. UV-Vis: *λ*_max,1_ = 317 nm (*ε*_1_ = 1.1 × 10^4^ L mol^−1^ cm^−1^, shoulder). Analysis calcd for C_55_H_55_LaN_2_P_2_Si_2_ [1001.08] C 65.99, H 5.54, N 2.80. Found: C 66.97, H 5.63, N 2.66.

#### Bis[1-(*N*-benzyldiphenylphosphazido)indenyl]bis(trimethyl-silyl)amide lanthanum 3-La

A vial equipped with a stirrer bar was charged with 1-La (449 mg, 0.500 mmol) and toluene (10 ml) and a solution of benzyl azide (134 mg, 1.00 mmol) in toluene (2 ml) was added dropwise under stirring. The mixture turned orange and was stirred for 4 h followed by evaporation of all volatiles. The red residue was taken up in a minimal amount of benzene, layered with hexane at ambient temperature to afford yellow crystals of 3-La (370 mg, 67%). mp 161 °C (dec). ^1^H NMR (*δ* in ppm, benzene-*d*6, 298 K): −0.01 (s, 18H, C*H*_3_), 4.87 (s, 4H, C*H*_2_), 5.61 (br, 2H, H2), 6.00 (br, 2H, H3), 6.91–7.06 (m, 16H, Ar–H), 7.07–7.14 (m, 10H, Ar–H), 7.16–7.20 (m, 2H, Ar–H), 7.61–7.79 (m, 10H, Ar–H). ^1^H NMR (*δ* in ppm, toluene-*d*8, 233 K): 0.00 (s, 18H, C*H*_3_), 4.58–4.86 (m, 2H), 4.88–5.08 (m, 4H), 5.73 (br, 1H), 6.66 (m, 1H, Ar–H), 6.55–6.86 (m, 5H), 6.90–7.04 (m, 12H), 7.05–7.09 (m, 3H), 7.19 (s, 3H), 7.24–7.34 (m, 3H), 7.39–7.50 (m, 3H), 7.53–7.61 (m, 1H), 7.63–7.89 (m, 8H), 8.27, (br, 1H). ^13^C{^1^H} NMR (*δ* in ppm, benzene-*d*6, 298 K): 3.9 (s, Si*C*H_3_), 64.7 (s, *C*H_2_), 73.4 (s, C1), 75.2 (s, C1), 109.5 (d, ^2^*J*_P,C_ = 14.0 Hz, C2), 119.8 (s, C_Ar_), 120.5 (s, C_Ar_), 120.8 (s, C_Ar_), 123.0 (s, C_Ar_), 127.6 (s, C_Ar_), 128.6 (d, *J*_P,C_ = 3.0 Hz, C_Ar_), 128.7 (s, C_Ar_), 128.8 (s, C_Ar_), 129.3 (d, *J*_P,C_ = 16.5 Hz, C_Ar_), 129.7 (s, C3), 130.0 (s, C_Ar_), 131.2 (s, C_Ar_), 132.0 (s, C_Ar_), 133.6 (d, *J*_P,C_ = 9.9 Hz, C_Ar_), 135.0 (d, *J*_P,C_ = 13.5 Hz, C_Ar_), 137.5 (s, C_Ar_). ^31^P{^1^H} NMR (*δ* in ppm, benzene-*d*6, 298 K): 16.5. ^31^P{^1^H} NMR (*δ* in ppm, toluene-*d*8, 233 K): 22.5, 9.7. UV-Vis: *λ*_max,1_ = 316 nm (*ε*_1_ = 1.6 × 10^4^ L mol^−1^ cm^−1^, shoulder), *λ*_max,2_ = 571 nm (*ε*_2_ = 3.7 × 10^2^ L mol^−1^ cm^−1^, broad band). Analysis calcd for C_62_H_64_LaN_7_P_2_Si_2_ [1164.27] C 63.96, H 5.54, N 8.42. Found: C65.11, H 5.52, N 8.08.

#### Bis[1-(*N*-benzyldiphenylphosphazido)indenyl]bis(trimethyl-silyl)amide samarium 3-Sm

The complex was prepared using the same procedure as for 3-La employing 1-Sm (898 mg, 1.00 mmol) and benzyl azide (269 mg, 2.00 mmol). Crystallization from benzene/pentane at ambient temperature yielded fine yellow needles of 3-Sm (606 mg, 52%). mp 166 °C (dec). ^1^H NMR (*δ* in ppm, benzene-*d*6, 298 K): −2.65 (s, 18 H, C*H*_3_), 5.35–9.39 (br, 49 H). ^1^H NMR (*δ* in ppm, toluene-*d*8, 233 K): −2.96 (s, 18H, C*H*_3_), 3.66 (d, 1H, *J*_H,H_ = 12.5 Hz), 3.81 (d, 1H, *J*_H,H_ = 6.5 Hz), 4.27 (d, 1H, *J*_H,H_ = 12.5 Hz), 4.79 (br, 1H), 5.95 (t, 2H *J*_H,H_ = 8.4 Hz) 6.16–6.35 (m, 6H), 6.43 (t, 2H, *J*_H,H_ = 7.2 Hz), 6.60 (t, 2H, *J*_H,H_ = 7.2 Hz), 6.69–6.90 (m, 6H), 6.99 (s, 2H), 7.07 (s, 2H), 7.33 (d, 1H, *J*_H,H_ = 7.4 Hz), 7.45 (t, 3 H, *J*_H,H_ = 7.4Hz), 7.72–7.83 (m, 4H), 7.94–8.11 (m, 3H), 8.22 (d, 2H, *J*_H,H_ = 7.4Hz), 8.56–8.73 (m, 2H), 10.11 (br, 2H), 10.42 (br, 2H), 13.02 (br, 1H). ^31^P{^1^H} NMR (*δ* in ppm, toluene-*d*8, 233 K): 18.9, 13.3. UV-Vis: *λ*_max,1_ = 318 nm (*ε*_1_ = 1.7 × 10^4^ L mol^−1^ cm^−1^, shoulder), *λ*_max,2_ = 555 nm (*ε*_2_ = 1.5 × 10^2^ L mol^−1^ cm^−1^, broad band). Analysis calcd for C_62_H_64_N_7_P_2_Si_2_Sm [1175.72] C 63.34, H 5.49, N 8.34. Found: C 64.29, H 5.13, N 7.73.

#### Typical procedure for the catalytic cyclisation/hydroamination of 2,2-diphenylpent-4-ene-1-amine using the presented 1-La, 1-Sm, 2, 3-La, or 3-Sm

Stock solutions of 2,2-diphenylpent-4-ene-1-amine and the catalyst were prepared in concentrations of 0.3 M and 0.015 M in benzene, respectively. In a standard procedure, a vial was charged with the appropriate amount of substrate (usually 1 mL), then 1 mL of benzene was added, obtaining a substrate concentration of 0.125 M after addition of the catalyst solution. The reaction was started by adding the catalyst solution (usually 0.4 mL) and samples were taken in specific time intervals in range of 3 to 15 min, depending on the conversion rates of the individual catalysts, to monitor the reaction progress. The samples taken were quenched on a suspension of (C_2_H_5_)_3_N·HCl in diethyl ether. The quenched samples were then analysed *via* GC-MS and the conversion obtained from the relative peak areas of the substrate and the hydroamination/cyclisation product.

## Conflicts of interest

There are no conflicts to declare.

## Supplementary Material

DT-051-D1DT03219D-s001

DT-051-D1DT03219D-s002

## References

[cit1] Schumann H., Meese-Marktscheffel J. A., Gorella B., Görlitz F. H. (1992). J. Organomet. Chem..

[cit2] Deacon G. B., Fallon G. D., Forsyth C. M. (1993). J. Organomet. Chem..

[cit3] Deacon G. B., Fallon G. D., Gatehouse B. M., Rabinovich A., Skelton B. W., White A. H. (1995). J. Organomet. Chem..

[cit4] Deacon G. B., Forsyth C. M., Gatehouse B. M., Philosof A., Skelton B. W., White A. H., White P. A. (1997). Aust. J. Chem..

[cit5] Lin G., Wong W.-T. (1995). Polyhedron.

[cit6] Lin G., Wong W.-T. (1995). J. Organomet. Chem..

[cit7] Lin G., Wong W.-T. (1996). J. Organomet. Chem..

[cit8] Lin G., Wong W.-T. (1994). Polyhedron.

[cit9] Deacon G., Forsyth C., Patalinghug W., White A., Dietrich A., Schumann H. (1992). Aust. J. Chem..

[cit10] Visseaux M., Dormond A., Kubicki M. M., Moïse C., Baudry D., Ephritikhine M. (1992). J. Organomet. Chem..

[cit11] Dormond A., Baudry D., Visseaux M., Hepiegne P. (1994). J. Alloys Compd..

[cit12] Deacon G. B., Dietrich A., Forsyth C. M., Schumann H. (1989). Angew. Chem..

[cit13] Broussier R., Delmas G., Perron P., Gautheron B., Petersen J. L. (1996). J. Organomet. Chem..

[cit14] Müller-Buschbaum K., Deacon G. B., Forsyth C. M. (2002). Eur. J. Inorg. Chem..

[cit15] Jian Z., Petrov A. R., Hangaly N. K., Li S., Rong W., Mou Z., Rufanov K. A., Harms K., Sundermeyer J., Cui D. (2012). Organometallics.

[cit16] Gololobov Yu. G., Zhmurova I. N., Kasukhin L. F. (1981). Tetrahedron.

[cit17] Liu B., Cui D. (2009). Dalton Trans..

[cit18] Broderick E. M., Thuy-Boun P. S., Guo N., Vogel C. S., Sutter J., Miller J. T., Meyer K., Diaconescu P. L. (2011). Inorg. Chem..

[cit19] Sun X., Su W., Shi K., Xie Z., Zhu C. (2020). Chem. – Eur. J..

[cit20] Dickie T. K. K., MacNeil C. S., Hayes P. G. (2020). Dalton Trans..

[cit21] Gagne M. R., Marks T. J. (1989). J. Am. Chem. Soc..

[cit22] Gagne M. R., Stern C. L., Marks T. J. (1992). J. Am. Chem. Soc..

[cit23] Giardello M. A., Conticello V. P., Brard L., Gagne M. R., Marks T. J. (1994). J. Am. Chem. Soc..

[cit24] Hong S., Kawaoka A. M., Marks T. J. (2003). J. Am. Chem. Soc..

[cit25] Müller T. E., Hultzsch K. C., Yus M., Foubelo F., Tada M. (2008). Chem. Rev..

[cit26] Curnow O. J., Fern G. M. (2002). Organometallics.

[cit27] Avent A. G., Edelman M. A., Lappert M. F., Lawless G. A. (1989). J. Am. Chem. Soc..

[cit28] Pangborn A. B., Giardello M. A., Grubbs R. H., Rosen R. K., Timmers F. J. (1996). Organometallics.

[cit29] Zanato C., Cascio M., Lazzari P., Pertwee R., Testa A., Zanda M. (2015). Synthesis.

[cit30] Schuetz S. A., Day V. W., Sommer R. D., Rheingold A. L., Belot J. A. (2001). Inorg. Chem..

[cit31] Hirner J. J., Roth K. E., Shi Y., Blum S. A. (2012). Organometallics.

[cit32] SAINTPLUS: Software Reference Manual, Version 6.45, Bruker-AXS, 1997–2003, Madison, WI

[cit33] Blessing R. H. (1995). Acta Crystallogr., Sect. A: Found. Crystallogr..

[cit34] Sheldrick G. M. , SADABS. Version 2.10, Bruker AXS Inc., Madison, WI, 2003

[cit35] Sheldrick G. M. (2008). Acta Crystallogr., Sect. A: Found. Crystallogr..

[cit36] Neese F. (2012). Wiley Interdiscip. Rev.: Comput. Mol. Sci..

[cit37] Neese F. (2018). Wiley Interdiscip. Rev.: Comput. Mol. Sci..

[cit38] Glendening E. D. , Badenhoop J. K., Reed A. E., Carpenter J. E., Bohmann J. A., Morales C. M., Karafiloglu P., Landis C. R. and Weinhold F., NBO 7.0, Theoretical Chemistry Institute, University of Wisconsin, Madison, 2018

[cit39] Rufanov K., Ziemer B., Hummert M., Schutte S. (2004). Eur. J. Inorg. Chem..

