# Supplementary material for: Phosphinoindenyl and phosphazidoindenyl complexes of lanthanum and samarium: synthesis, characterisation, and hydroamination catalysis
Source: Dalton Trans. 2021 Dec 24;51(5):1819–28. doi: 10.1039/d1dt03219d (PMC8805144; doi:10.1039/d1dt03219d)
Supplement: DT-051-D1DT03219D-s001 [file DT-051-D1DT03219D-s001.pdf]

Electronic Supplementary Material (ESI) for Dalton Transactions.
This journal is © The Royal Society of Chemistry 2021

Supplementary Information

Phosphinoindenyl and Phosphazidoindenyl Complexes of Lanthanum and Samarium: Synthesis, Characterisation, and Hydroamination Catalysis

Matthias R. Steiner,[a] *Johann A. Hlina,**[a] *Johanna M. Uher,*[a] *Roland C. Fischer,*[a] *Dmytro Neshchadin,*[b] *and Theresa Wilfling*[a]

[a] Institute of Inorganic Chemistry, Graz University of Technology, Stremayrgasse 9, 8010 Graz, Austria.
[b] Institute of Physical and Theoretical Chemistry, Graz University of Technology, Stremayrgasse 9, 8010 Graz, Austria.

Table of Contents

Molecular Structures: 2
Crystallographic Data: 3
Hydroamination Catalysis 5
NMR Spectroscopy 8
UV-Vis Spectroscopy 21
Computational Data 22

Molecular Structures:

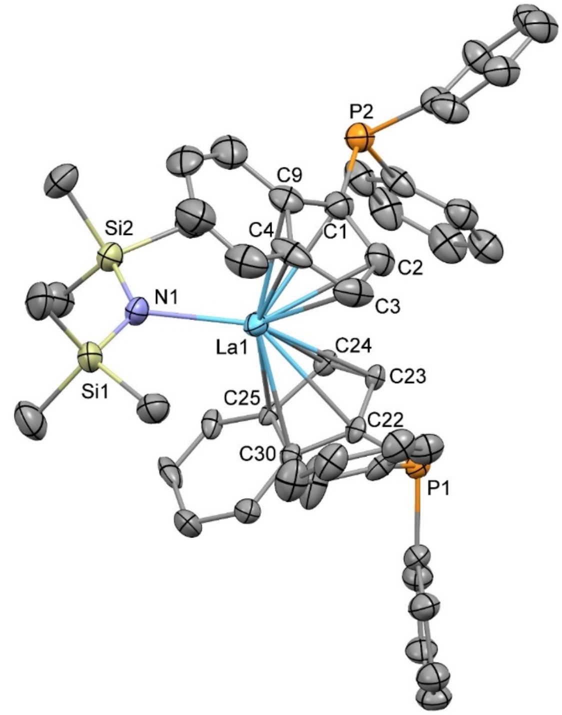

Figure S1. Molecular structure of **1-La**. Hydrogen atoms are omitted for clarity. Thermal ellipsoids drawn at 50 % probability. Selected distances (Å) and angles (deg): La1-N1: 2.339(3), La1-C1: 2.842(4), La1-C2: 2.851(5), La1-C3: 2.822(5), La1-C4: 2.835(5), La1-C9: 2.859(4), La1-C22: 2.89(2), La1-C23: 2.910(9), La1-C24: 2.861(8), La1-C25: 2.85(1), La1-C30: 2.88(1), C22-P1: 1.83(2), C1-P2: 1.807(4), La1-N1-Si1: 116.5(2), La1-N1-Si2: 114.0(2).

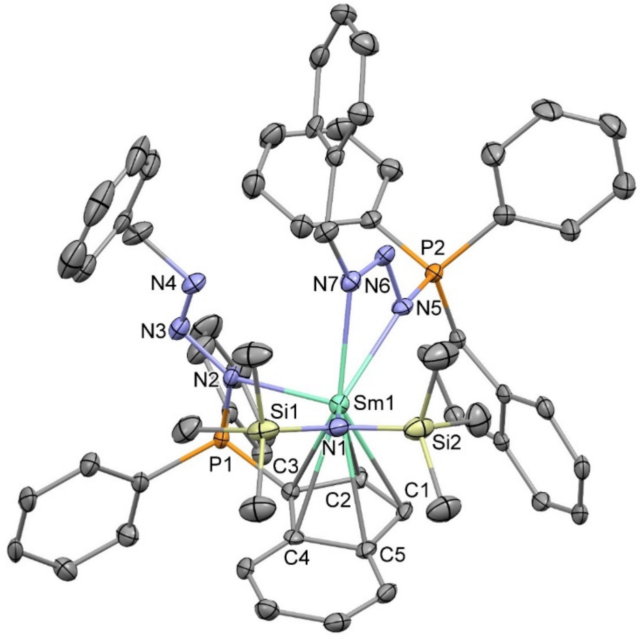

Figure S2. Molecular structure of **3-Sm**. Hydrogen atoms are omitted for clarity. Thermal ellipsoids drawn at 50 % probability. Selected distances (Å) and angles (deg): Sm1-N1: 2.266(4), Sm1-N2: 2.496(4), Sm1-N5: 2.534(4), Sm1-N7: 2.523(5), N2-N3: 1.397(6), N3-N4: 1.248(6), N5-N6: 1.380(6), N6-N7: 1.273(4), P1-N2: 1.638(4), P2-N5: 1.676(3), Sm1-C1: 2.874(5), Sm1-C2: 2.777(4), Sm1-C3: 2.739(5), Sm1-C4: 2.846(6), Sm1-C5: 2.934(6), C3-P1: 1.751(4), C29-P2: 1.717(5), N1-Sm1-N2: 120.6(1), N5-Sm1-N7: 50.6(1), N2-N3-N4: 111.2(4), N5-N6-N7: 108.9(4).

Crystallographic Data:

Table S1. Crystallographic data of the complexes **1-La**, **1-Sm**, and **2**.

	**1-La**	**1-Sm**	**2**
CCDC number	2108459	2108458	2108460
Empirical formula	$C_{48}H_{50}LaNP_2Si_2$	$C_{48}H_{50}NP_2Si_2Sm$	$C_{55}H_{55}LaN_2P_2Si_2$
Formula weight	897.92	909.36	1001.04
Temperature /K	99.98	100(2)	150(2)
Crystal system	triclinic	monoclinic	monoclinic
Space group	*P*-1	$P2_1/n$	*C*2/*c*
a /Å	9.6450(6)	9.9293(19)	48.469(10)
b /Å	11.8885(6)	37.125(7)	13.677(3)
c /Å	19.4700(10)	11.726(2)	18.852(4)
α /°	79.178(3)	90	90
β /°	85.972(3)	95.440(3)	112.195(5)
γ /°	82.708(3)	90	90
Volume /Å^3	2172.6(2)	4303.0(14)	11571(4)
Z	2	4	8
ρ_{calc}g /cm^3	1.373	1.404	1.149
M /mm^{-1}	1.145	1.529	0.867
F(000)	920.0	1860	4112
Crystal size /mm^3	0.17 x 0.14 x 0.10	0.45 x 0.14 x 0.10	0.37 x 0.30 x 0.22
2Θ range for data collection /°	2.132 to 53.996	1.83 to 26.37	2.72 to 26.35
Index ranges	-12<=h<=12, -15<=k<=15, -24<=l<=24	-12<=h<=12, -46<=k<=46, -14<=l<=14	-58<=h<=60, -17<=k<=17, -23<=l<=23
Reflections collected	114466	34193	45163
Independent reflections	9498 [R(int) = 0.0655]	8807 [R(int) = 0.0371]	11798 [R(int) = 0.0409]
Data/restraints/parameters	9498 / 438 / 692	8807 / 0 / 493	11798 / 39 / 566
Goodness-of-fit on F^2	1.141	1.218	1.077
Final R indexes [I>=2σ (I)]	R1 = 0.0512, wR2 = 0.1110	R1 = 0.0433, wR2 = 0.0919	R1 = 0.0378, wR2 = 0.0918
Final R indexes [all data]	R1 = 0.0618, wR2 = 0.1168	R1 = 0.0465, wR2 = 0.0934	R1 = 0.0422, wR2 = 0.0943
Largest diff. peak/hole / e Å^{-3}	3.19 and -1.06	1.772 and -1.095	1.536 and -0.492

Table S2. Crystallographic data of the complexes **3-La** and **3-Sm**.

	**3-La**	**3-Sm**
CCDC number	2109013	2110760
Empirical formula	$C_{69}H_{72}LaN_7P_2Si_2$	$C_{69}H_{72}N_7P_2Si_2Sm$
Formula weight	1164.23	1175.67
Temperature /K	150(2)	99.99
Crystal system	triclinic	triclinic
Space group	*P*-1	*P*-1
a /Å	12.632(3)	12.6691(13)
b /Å	13.456(3)	13.4210(13)
c /Å	21.811(5)	21.6089(17)
α /°	100.696(4)	100.322(5)
β /°	97.570(4)	97.953(5)
γ /°	115.177(3)	115.115(5)
Volume /$Å^3$	3203.0(12)	3176.8(5)
Z	2	2
ρ_{calc}g /cm^3	1.303	1.325
M /mm^{-1}	0.800	1.059
F(000)	1300	1310
Crystal size /mm^3	0.34 x 0.15 x 0.09	0.34 x 0.20 x 0.05
2Θ range for data collection /°	1.736 to 26.427	1.756 to 28.000
Index ranges	-15<=h<=15,<br>-16<=k<=16,<br>-27<=l<=27	-16<=h<=16,<br>-17<=k<=17,<br>-28<=l<=28
Reflections collected	25997	55667
Independent reflections	12976 [R(int) = 0.1022]	12050 [R(int) = 0.0835]
Data/restraints/parameters	12976 / 0 / 673	14510 / 0 / 673
Goodness-of-fit on F^2	1.175	1.058
Final R indexes [I>=2σ (I)]	R1 = 0.0742, wR2 = 0.1903	R1 = 0.0526, wR2 = 0.1003
Final R indexes [all data]	R1 = 0.0847, wR2 = 0.1967	R1 = 0.0710, wR2 = 0.1084
Largest diff. peak/hole / e $Å^{-3}$	1.927 and -1.364	1.89 and -1.34

Hydroamination Catalysis:

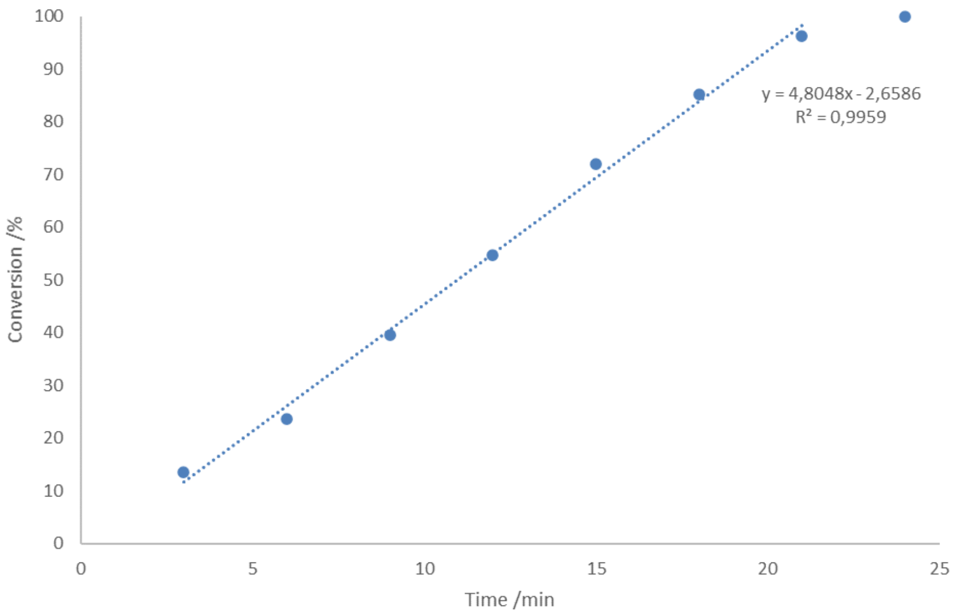

Figure S3. Conversion rates of the hydroamination/cyclisation of 2,2-diphenylpent-4-ene-1-amine using 2 mol% of **1-La** as catalyst at ambient temperature in benzene. The trend line is drawn as dotted line and excludes the full conversion data point.

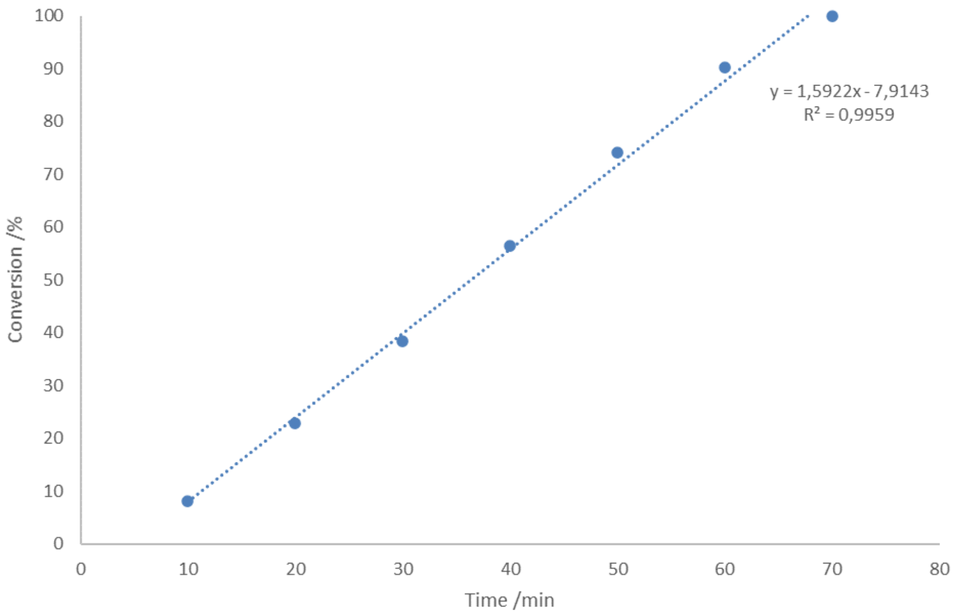

Figure S4. Conversion rates of the hydroamination/cyclisation of 2,2-diphenylpent-4-ene-1-amine using 2 mol% of **1-Sm** as catalyst at ambient temperature in benzene. The trend line is drawn as dotted line and excludes the full conversion data point.

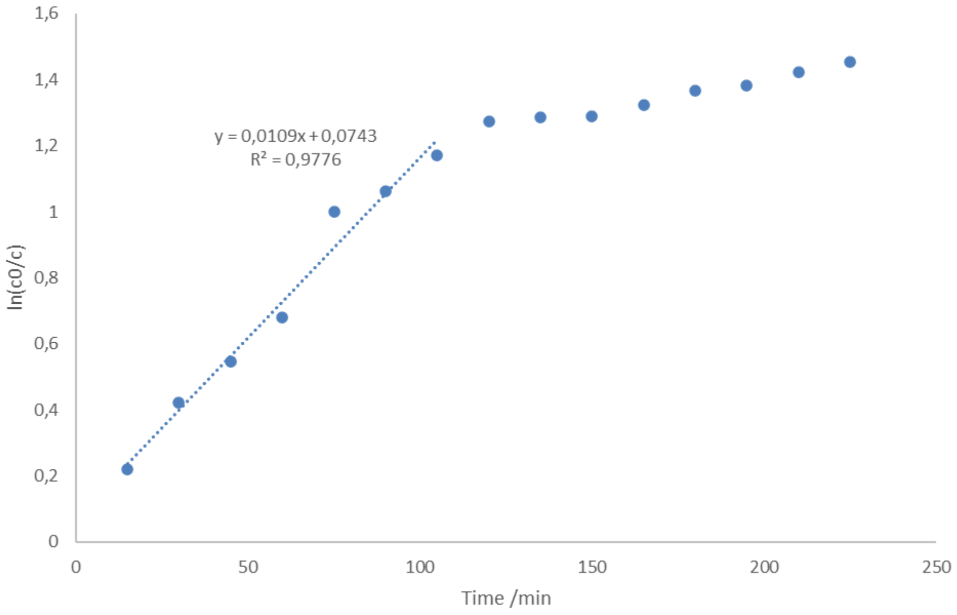

Figure S5. The ln(c0/c) (c0 = initial substrate concentration, c = substrate concentration at certain reaction time) versus reaction time for the hydroamination/cyclisation of 2,2-diphenylpent-4-ene-1-amine using 2 mol% of **2** as catalyst at ambient temperature in benzene. The trend line is drawn as dotted line for early reaction phase only.

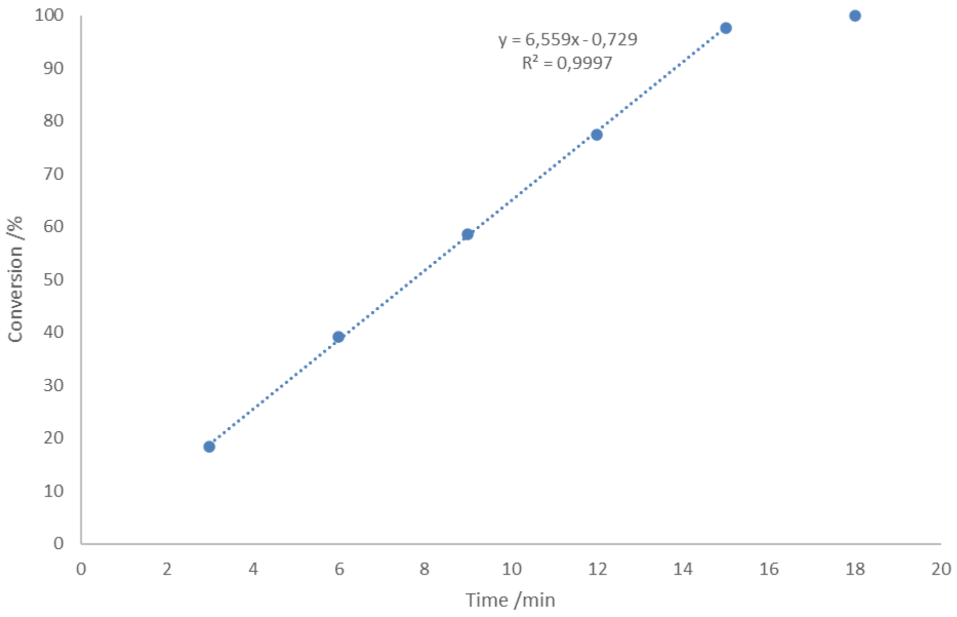

Figure S6. Conversion rates of the hydroamination/cyclisation of 2,2-diphenylpent-4-ene-1-amine using 2 mol% of **3-La** as catalyst at ambient temperature in benzene. The trend line is drawn as dotted line and excludes the full conversion data point.

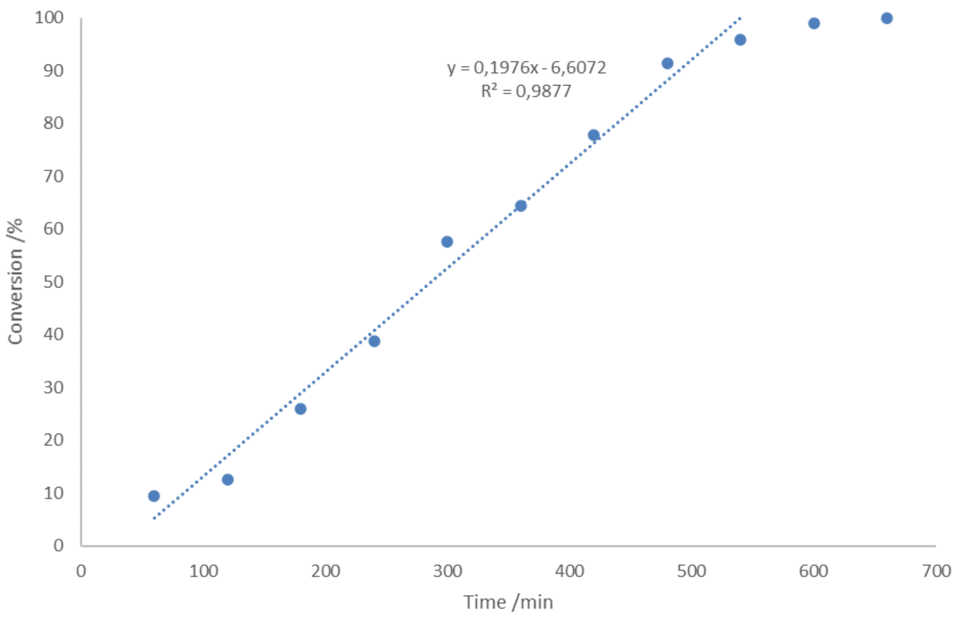

Figure S7. Conversion rates of the hydroamination/cyclisation of 2,2-diphenylpent-4-ene-1-amine using 2 mol% of **3-Sm** as catalyst at ambient temperature in benzene. The trend line is drawn as dotted line and excludes the last two data points nearing full conversion.

NMR Spectroscopy:

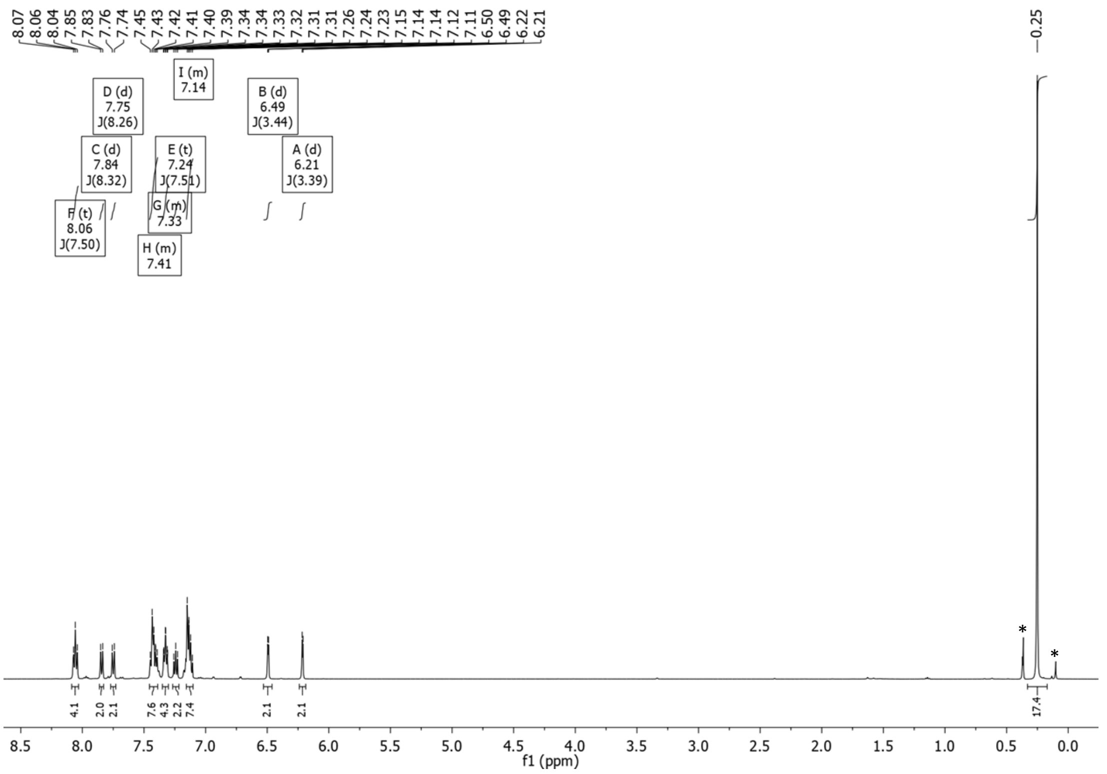

Figure S8. ^{1}H NMR spectrum of **1-La** recorded from a solution in benzene-d6 at 298 K. Trace impurities are marked with an asterisk.

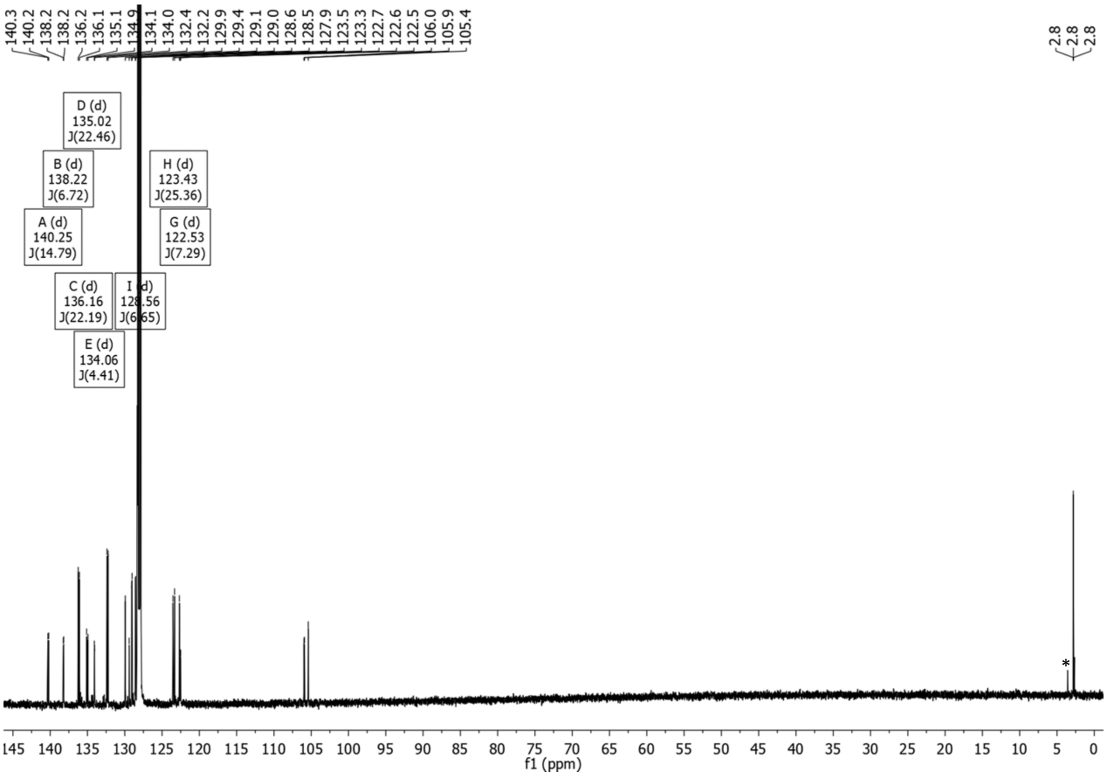

Figure S9. $^{13}C\{^{1}H\}$ NMR spectrum of **1-La** recorded from a solution in benzene-d6 at 298 K. Trace impurities are marked with an asterisk.

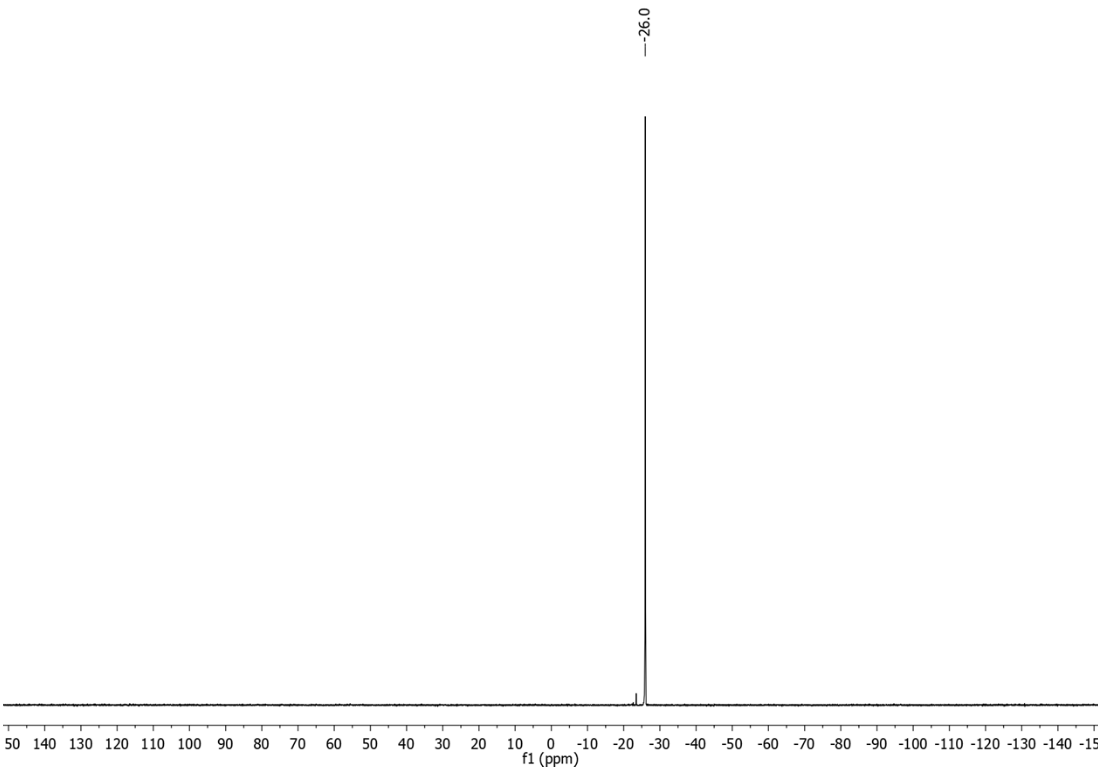

Figure S10. $^{31}P\{^{1}H\}$ NMR spectrum of **1-La** recorded from a solution in benzene-d6 at 298 K.

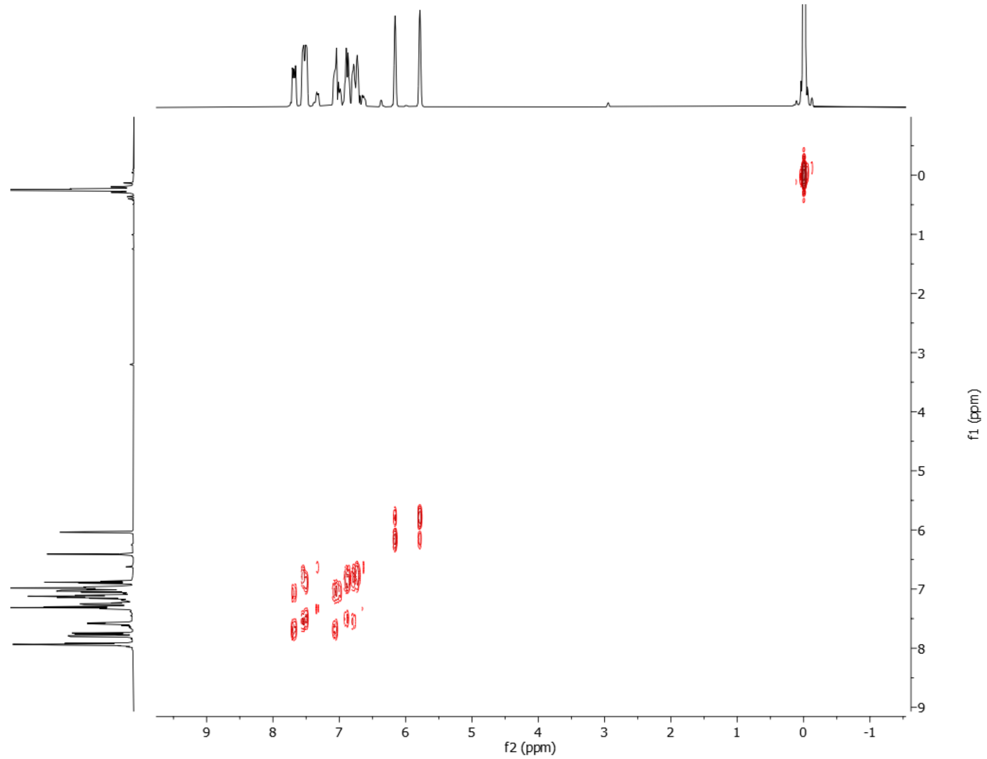

Figure S11. ^{1}H-^{1}H COSY NMR spectrum of **1-La** recorded from a solution in benzene-d6 at 298 K.

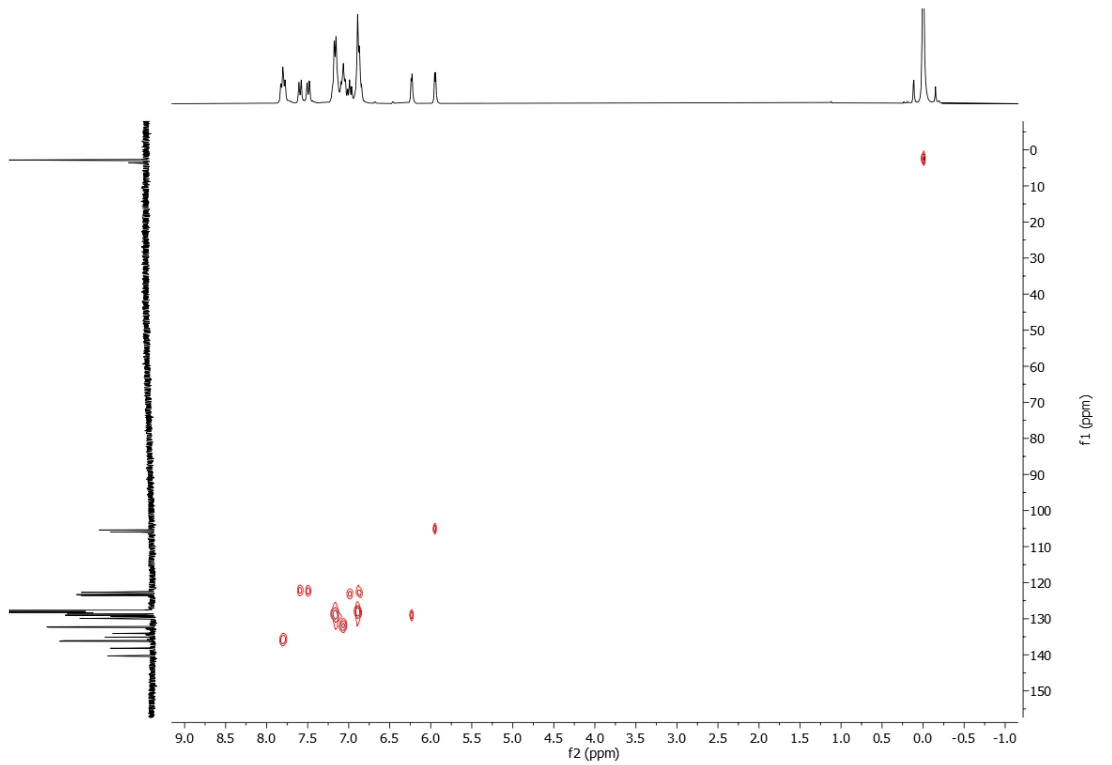

Figure S12. ^{1}H-^{13}C HSQC NMR spectrum of **1-La** recorded from a solution in benzene-d6 at 298 K.

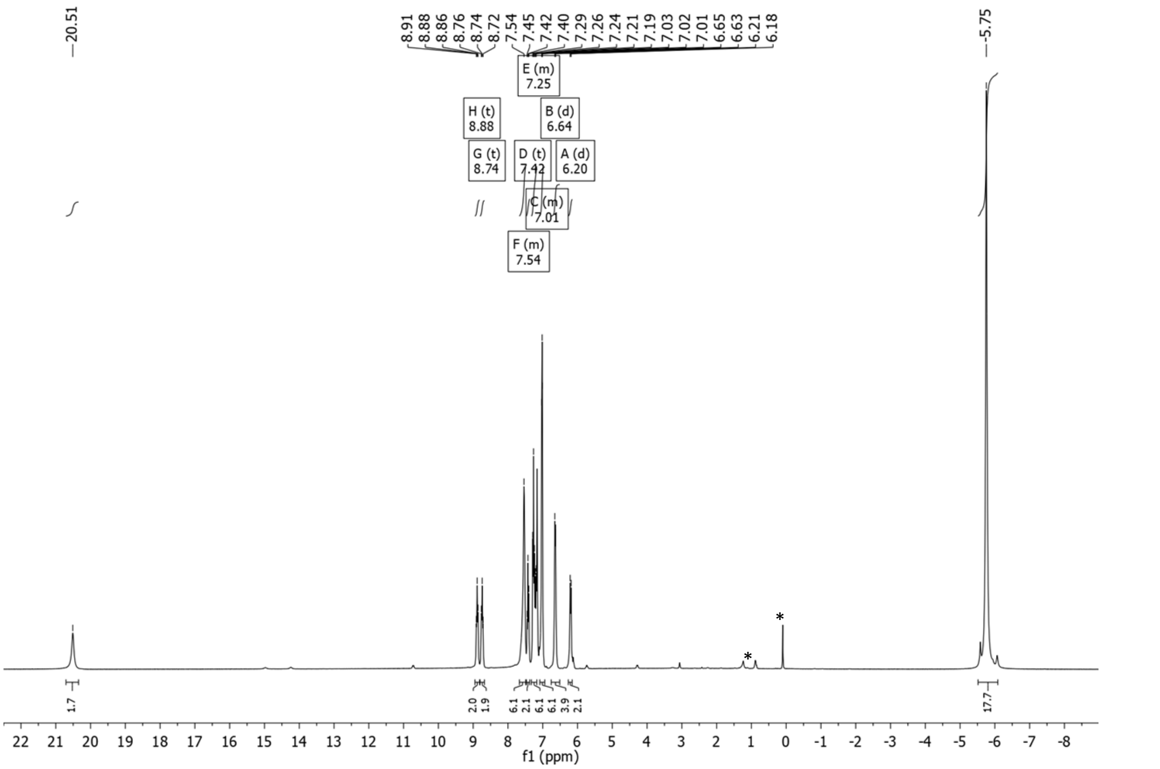

Figure S13. ^{1}H NMR spectrum of **1-Sm** recorded from a solution in benzene-d6 at 298 K. Trace impurities are marked with an asterisk.

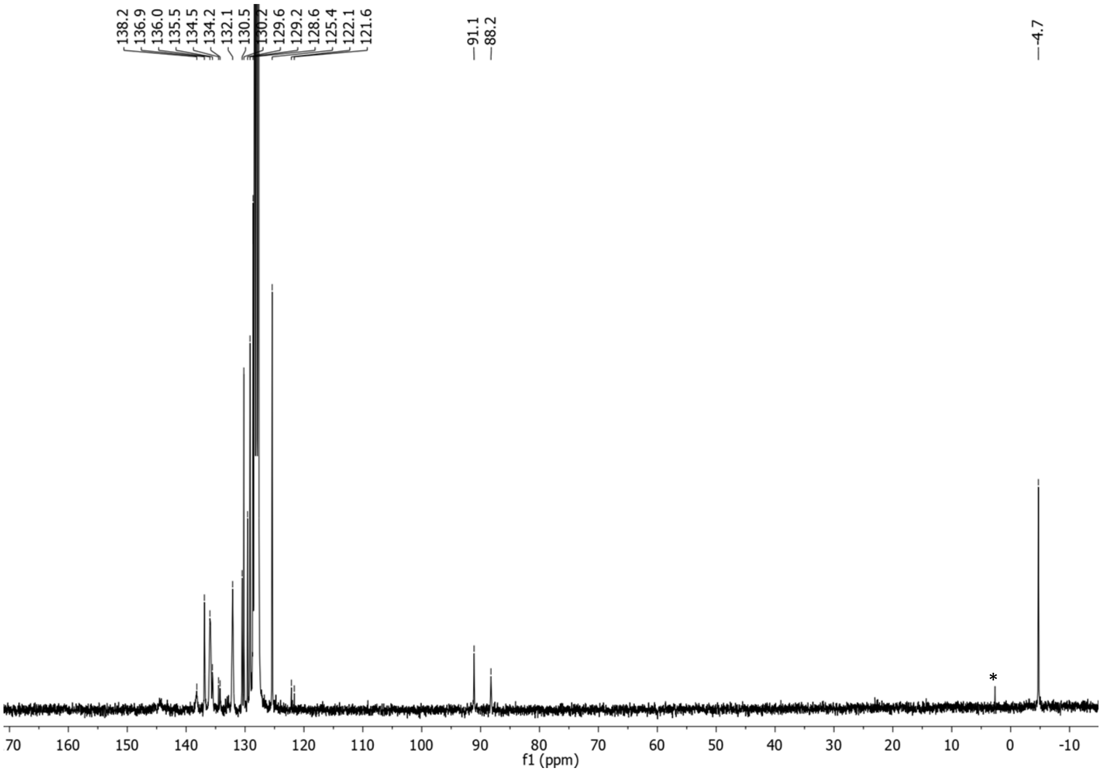

Figure S14. ${}^{13}C\{{}^{1}H\}$ NMR spectrum of **1-Sm** recorded from a solution in benzene-d6 at 298 K. Trace impurities are marked with an asterisk.

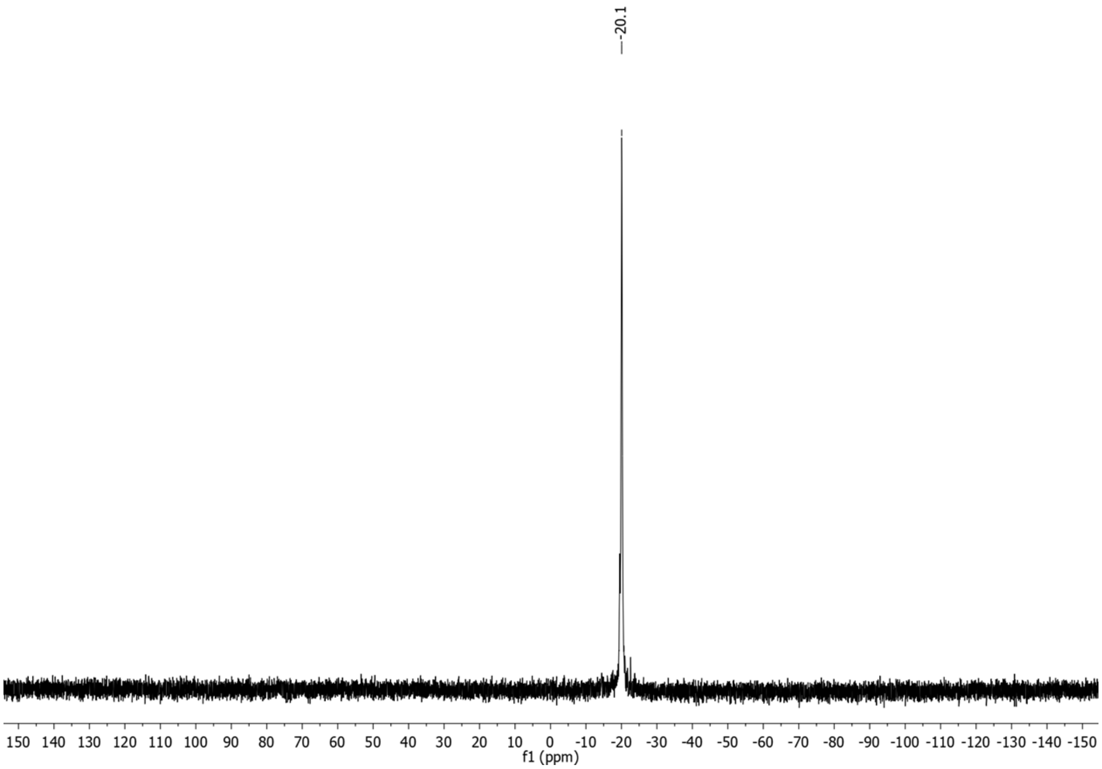

Figure S15. ${}^{31}P\{{}^{1}H\}$ NMR spectrum of **1-Sm** recorded from a solution in benzene-d6 at 298 K.

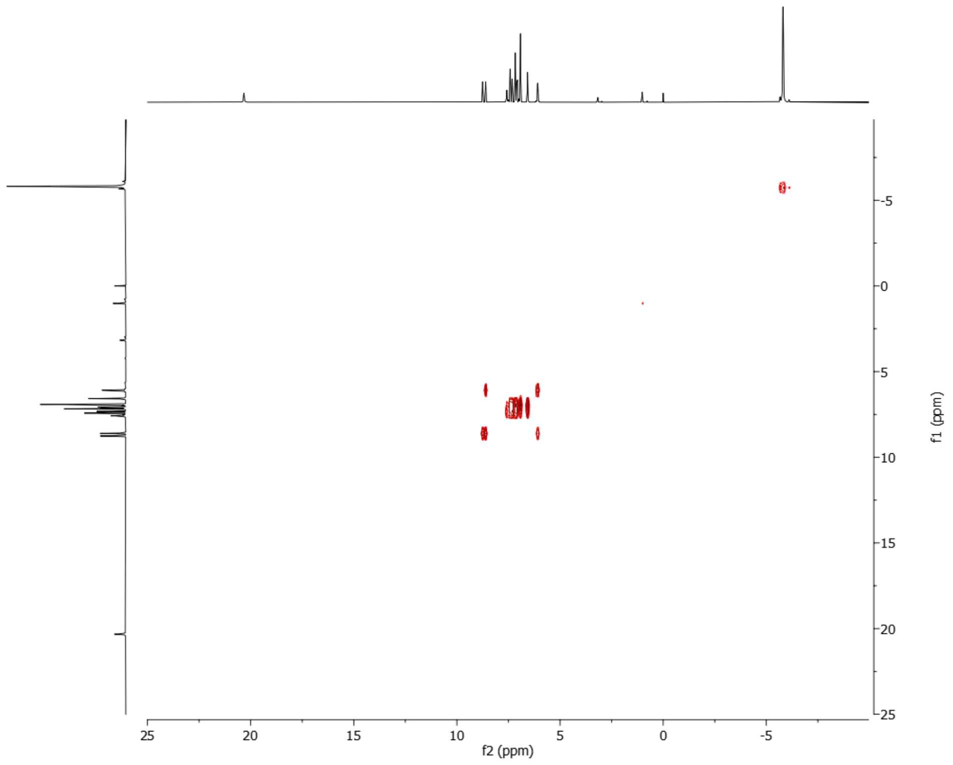

Figure S16. ^{1}H-^{1}H COSY NMR spectrum of **1-Sm** recorded from a solution in benzene-d6 at 298 K.

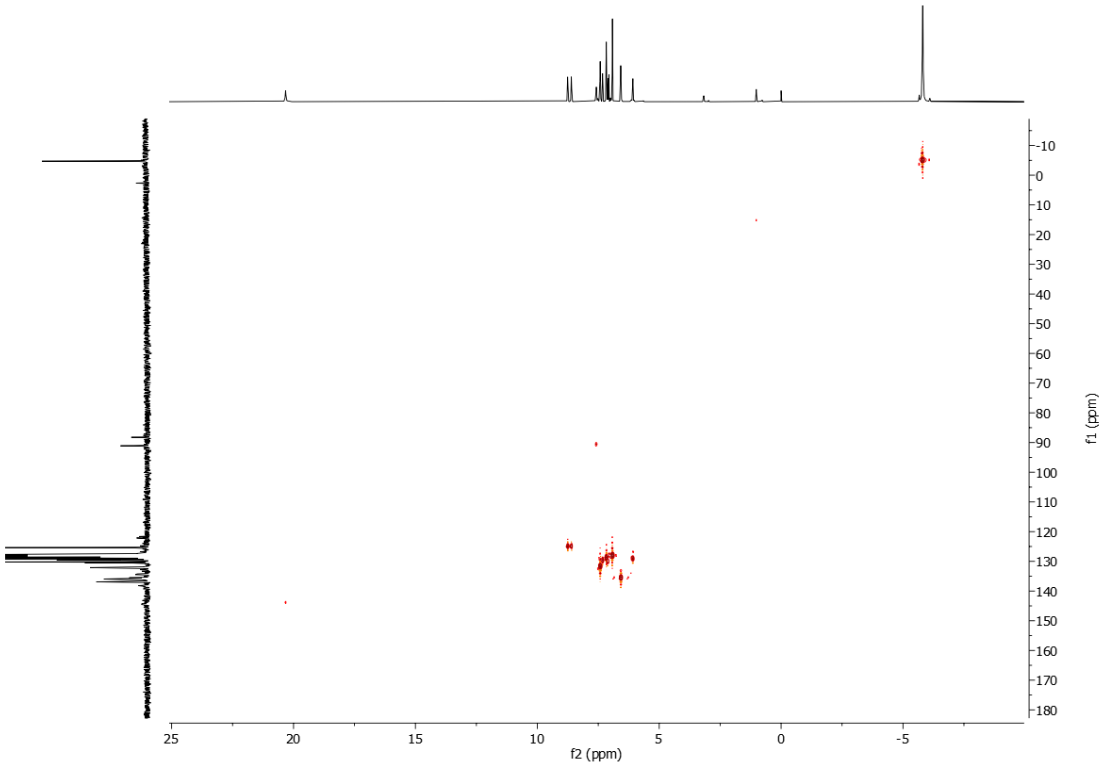

Figure S17. ^{1}H-^{13}C HSQC NMR spectrum of **1-La** recorded from a solution in benzene-d6 at 298 K.

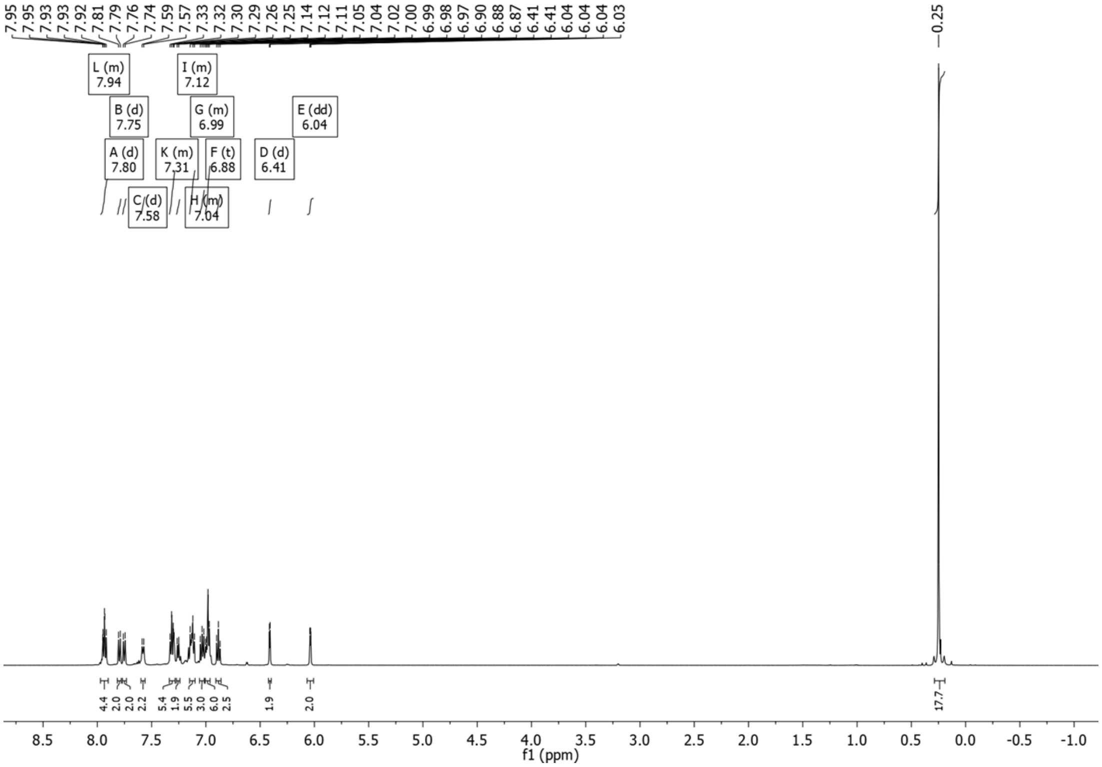

Figure S18. ^{1}H NMR spectrum of **2** recorded from a solution in benzene-d6 at 298 K.

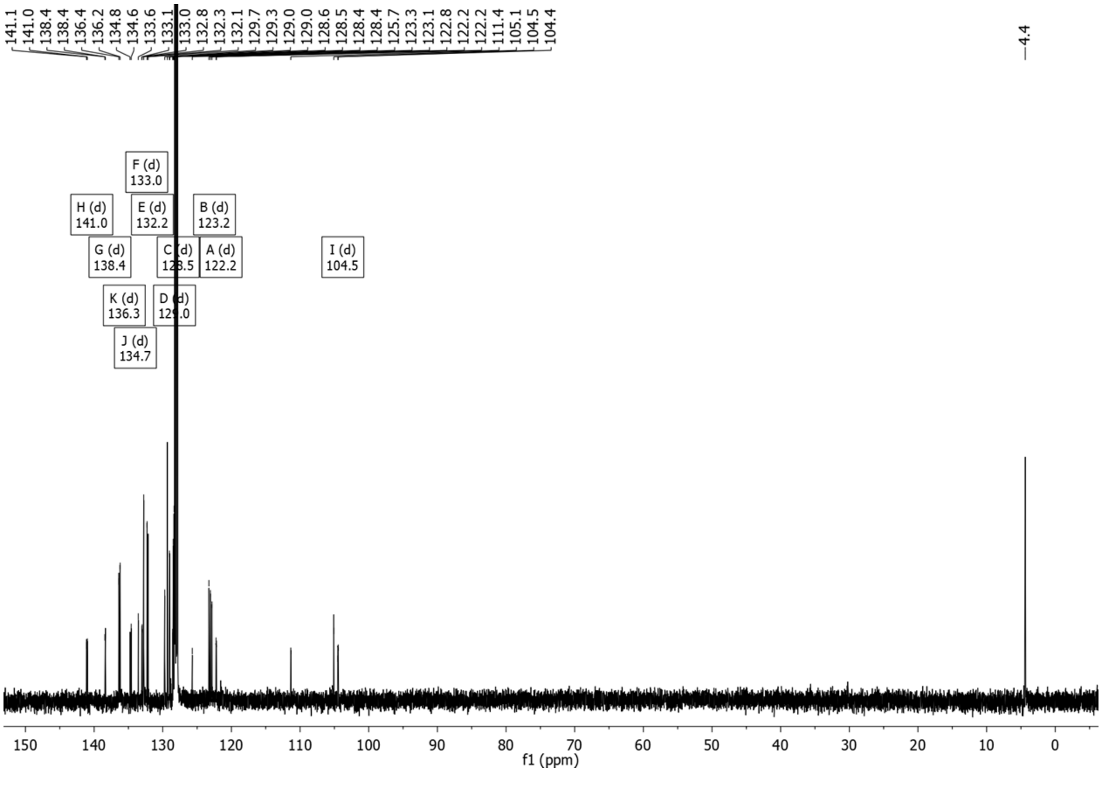

Figure S19. $^{13}C\{^{1}H\}$ NMR spectrum of **2** recorded from a solution in benzene-d6 at 298 K.

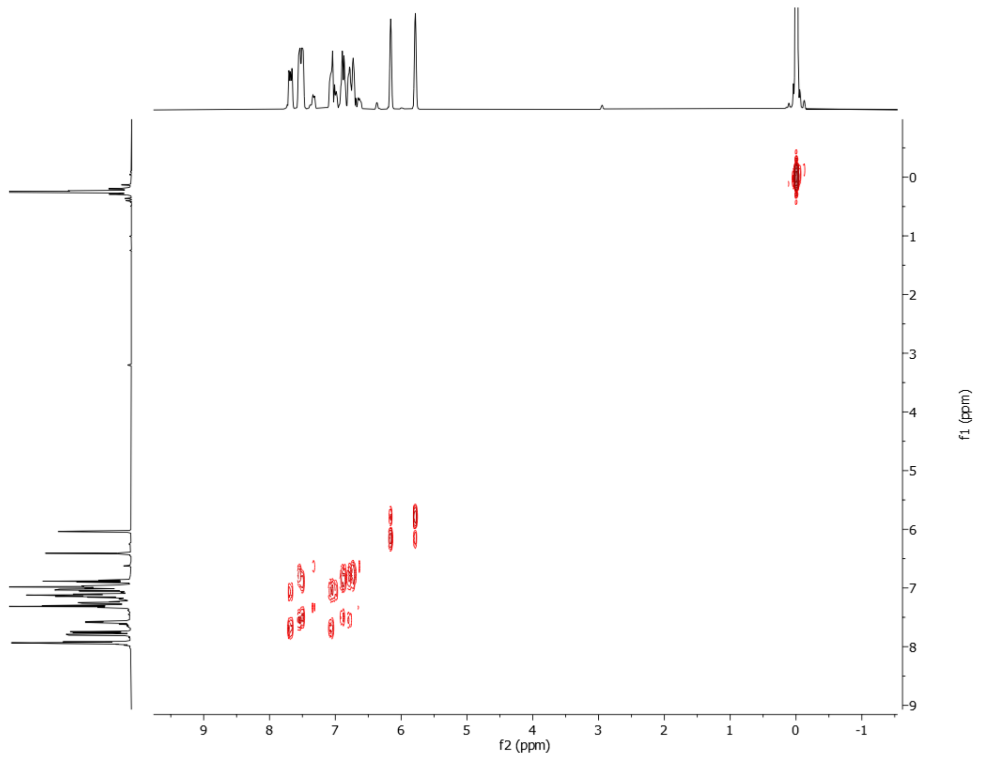

Figure S20. ^{1}H-^{1}H COSY NMR spectrum of **2** recorded from a solution in benzene-d6 at 298 K.

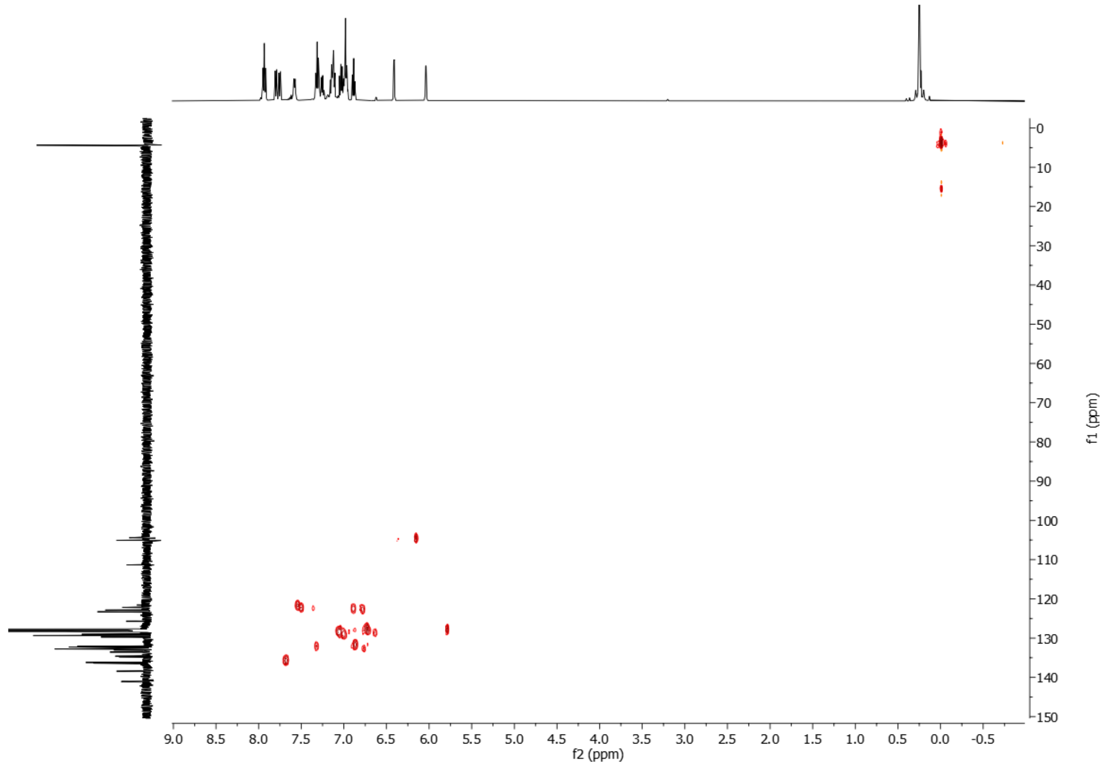

Figure S21. ^{1}H-^{13}C HSQC NMR spectrum of **1-La** recorded from a solution in benzene-d6 at 298 K.

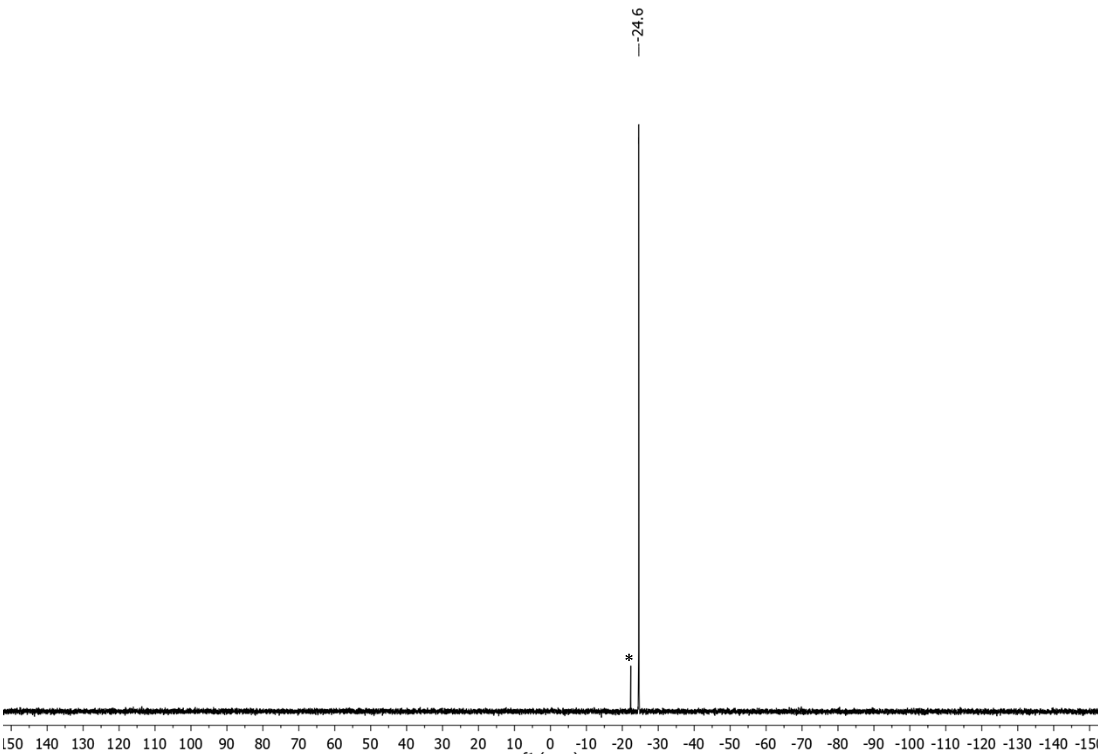

Figure S22. $^{31}P\{^{1}H\}$ NMR spectrum of **2** recorded from a solution in benzene-d6 at 298 K. Trace impurities are marked with an asterisk.

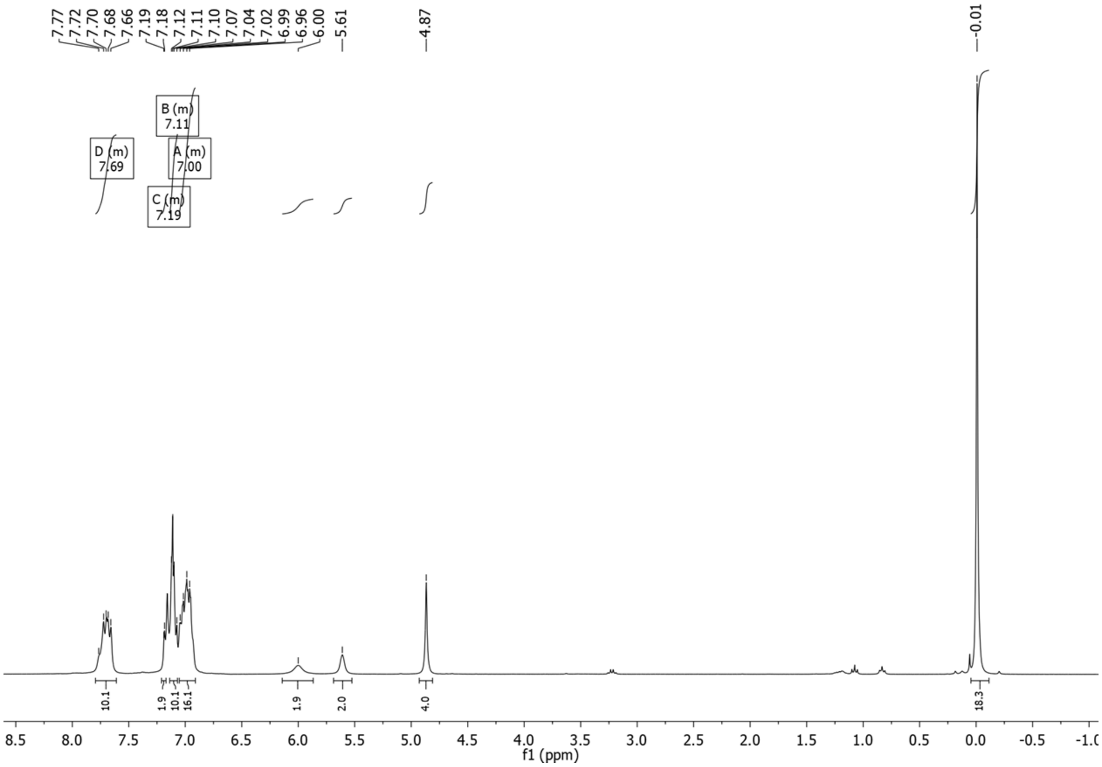

Figure S23. ^{1}H NMR spectrum of **3-La** recorded from a solution in benzene-d6 at 298 K.

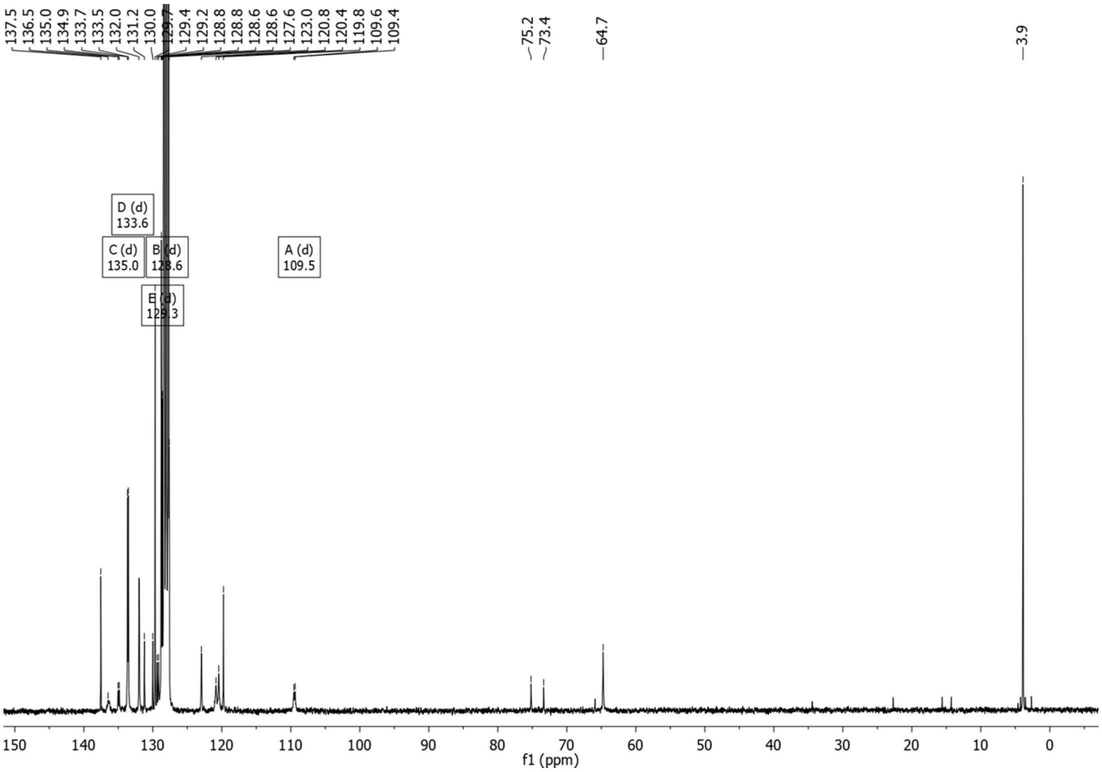

Figure 24. $^{13}C\{^{1}H\}$ NMR spectrum of **3-La** recorded from a solution in benzene-d6 at 298 K.

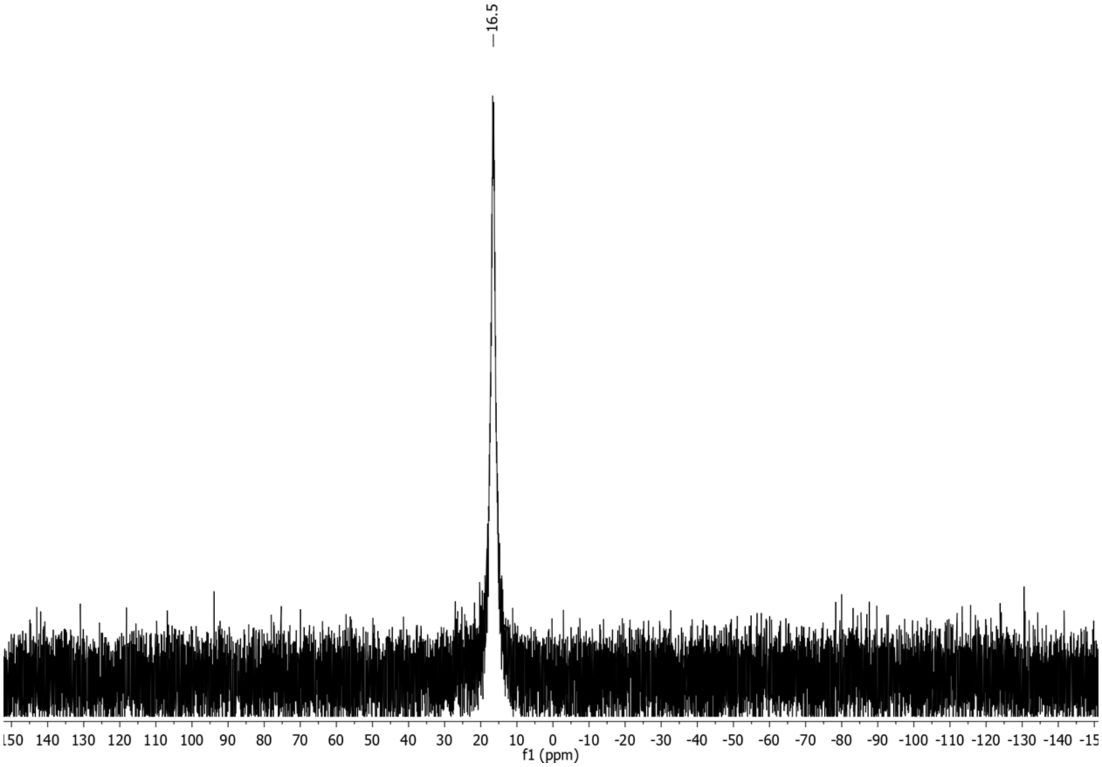

Figure S25. $^{31}P\{^{1}H\}$ NMR spectrum of **3-La** recorded from a solution in benzene-d6 at 298 K.

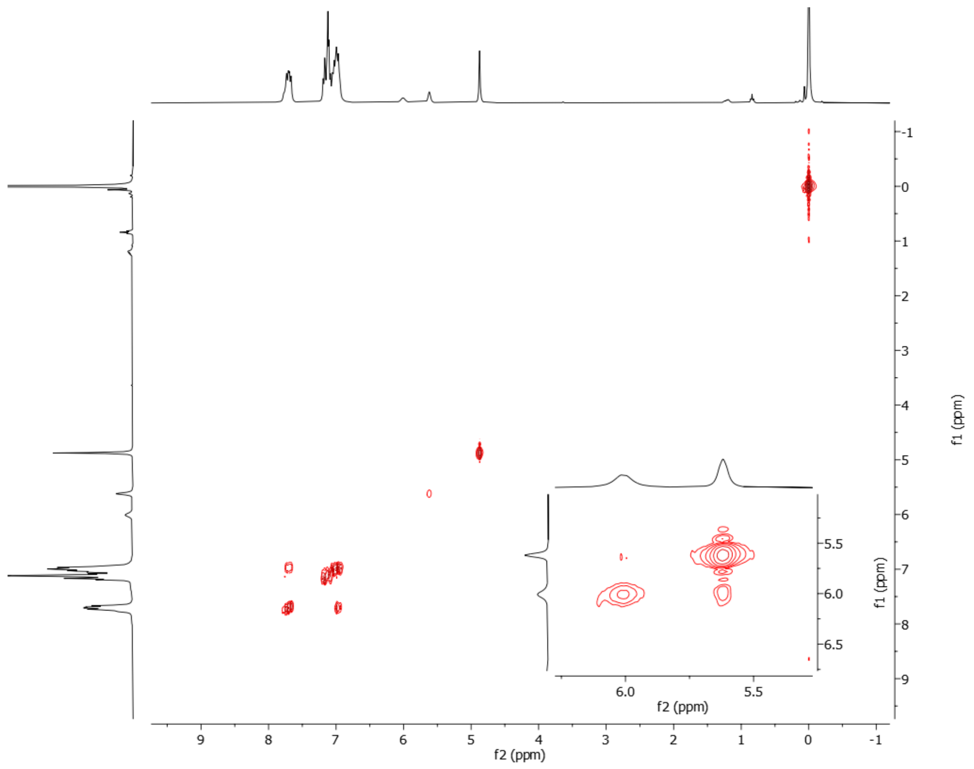

Figure S26. ¹H-¹H COSY NMR spectrum of **3-La** recorded from a solution in benzene-d6 at 298 K.

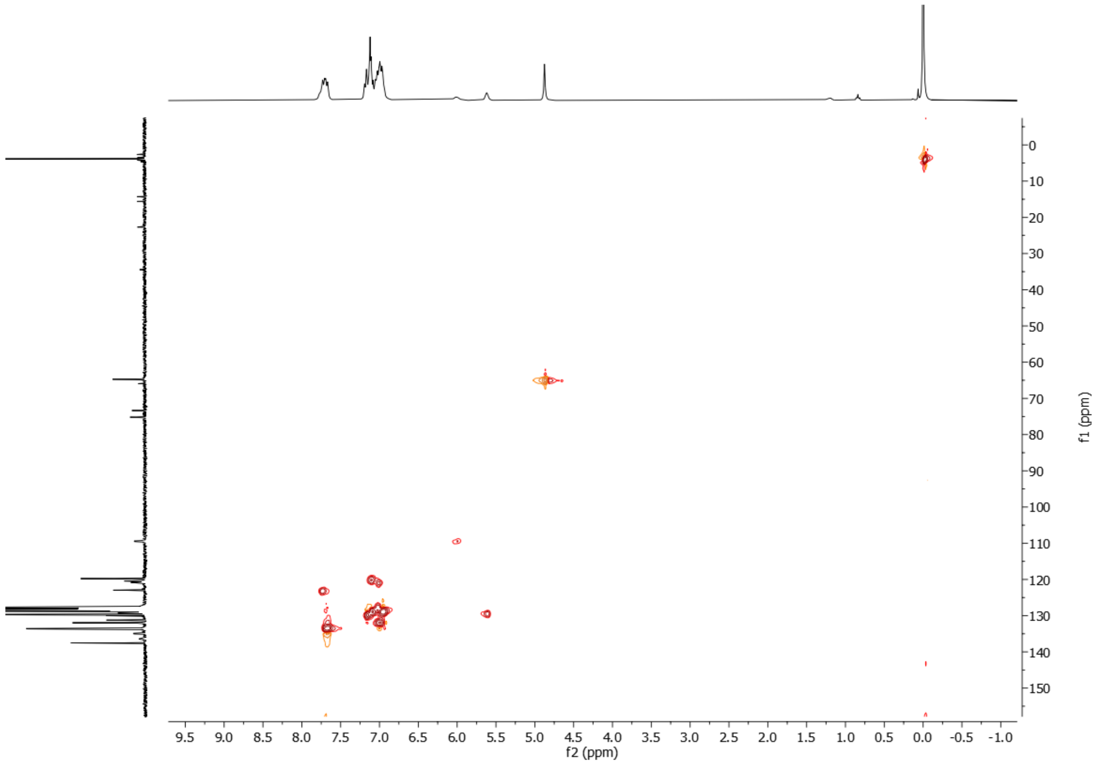

Figure S27. ¹H-¹³C HSQC NMR spectrum of **3-La** recorded from a solution in benzene-d6 at 298 K.

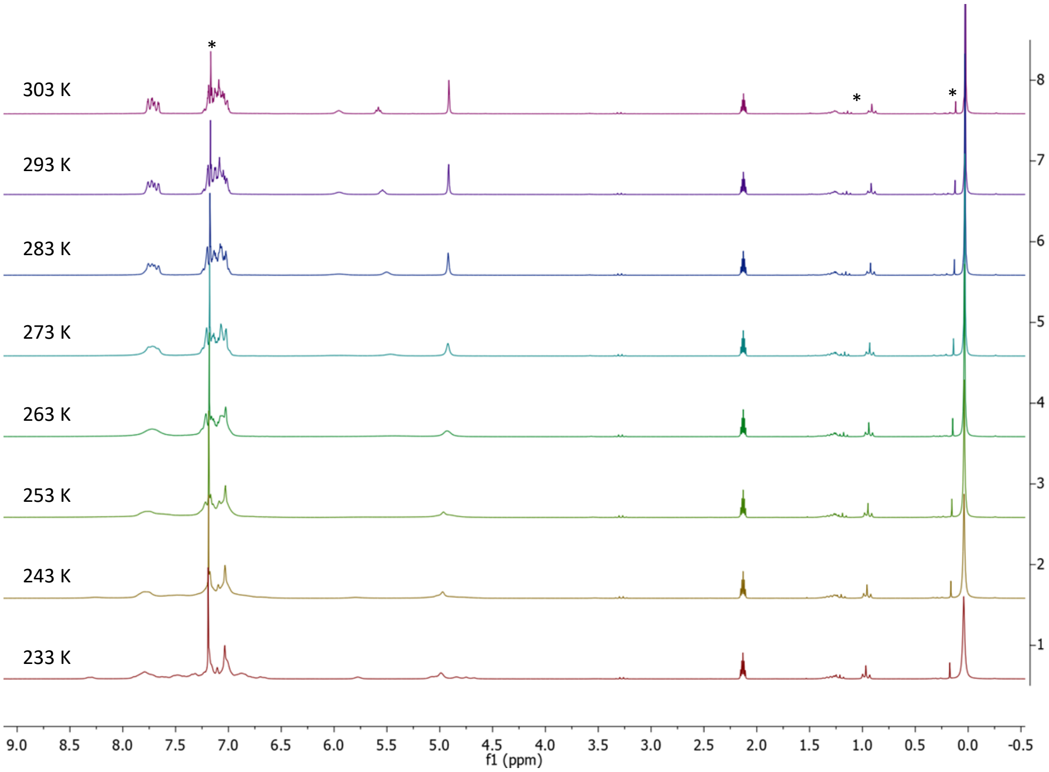

Figure S28. ^{1}H VT NMR spectra of **3-La** recorded from a solution in toluene-d8 from 233 to 303 K in 10 K intervals. Trace impurities are marked with an asterisk.

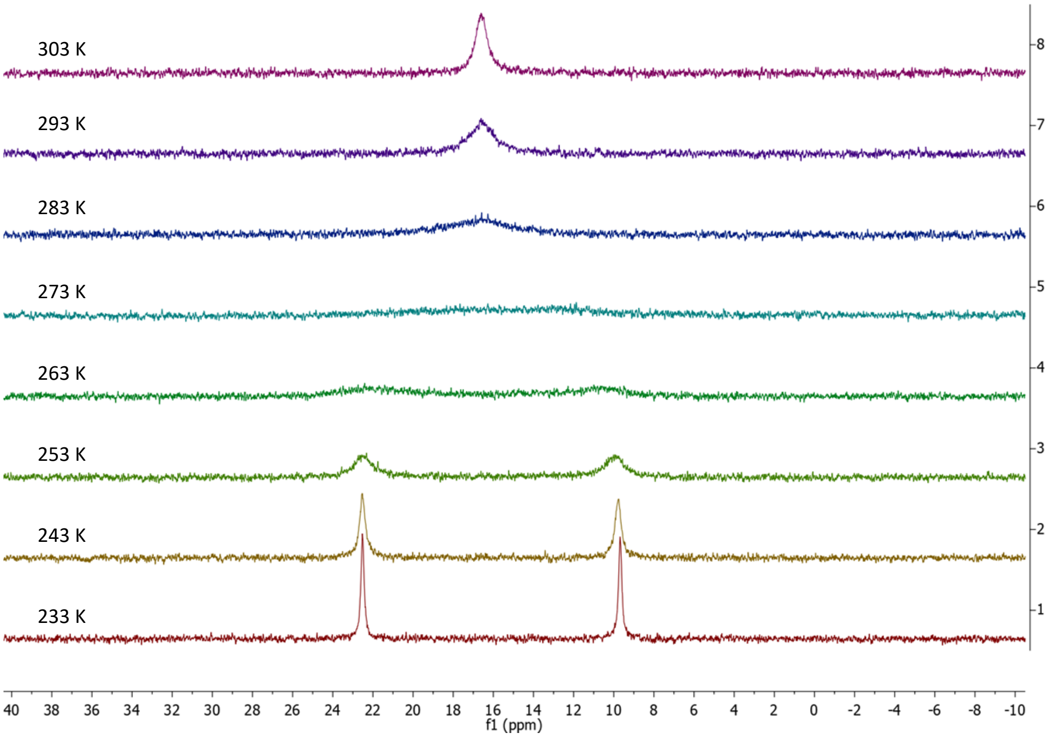

Figure S29. $^{31}P\{^{1}H\}$ VT NMR spectra of **3-La** recorded from a solution in toluene-d8 from 233 to 303 K in 10 K intervals.

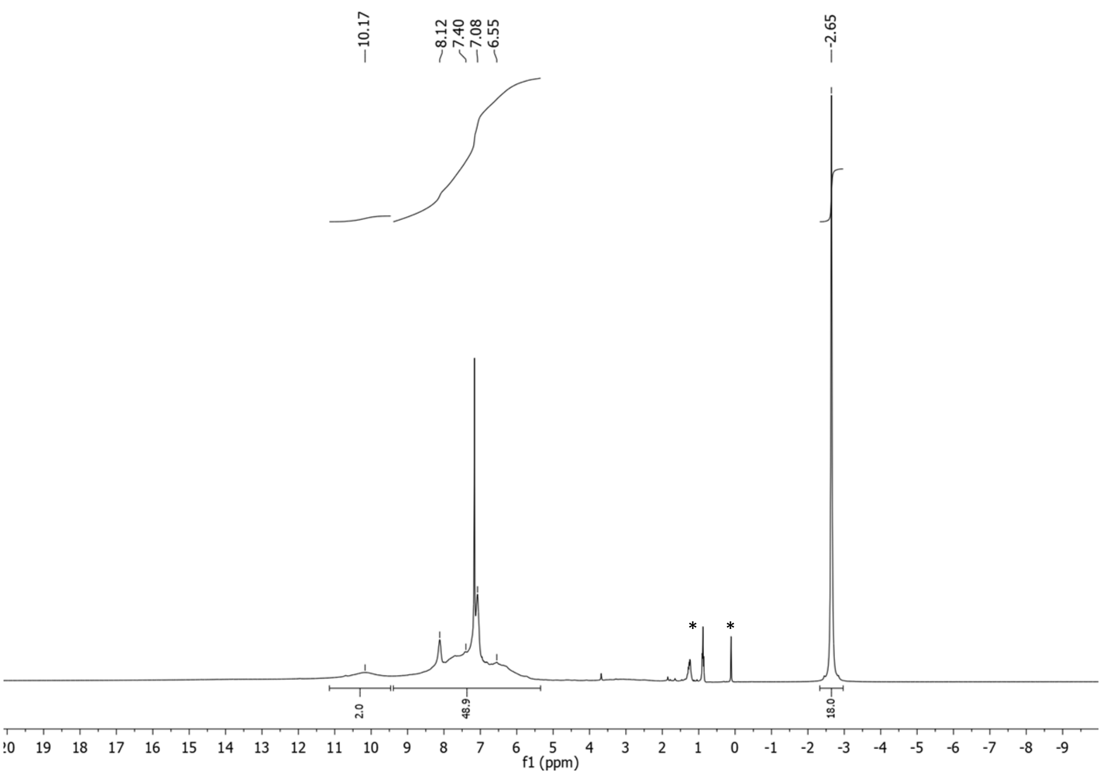

Figure S30. ^{1}H NMR spectrum of **3-Sm** recorded from a solution in benzene-d6 at 298 K. Trace impurities are marked with an asterisk.

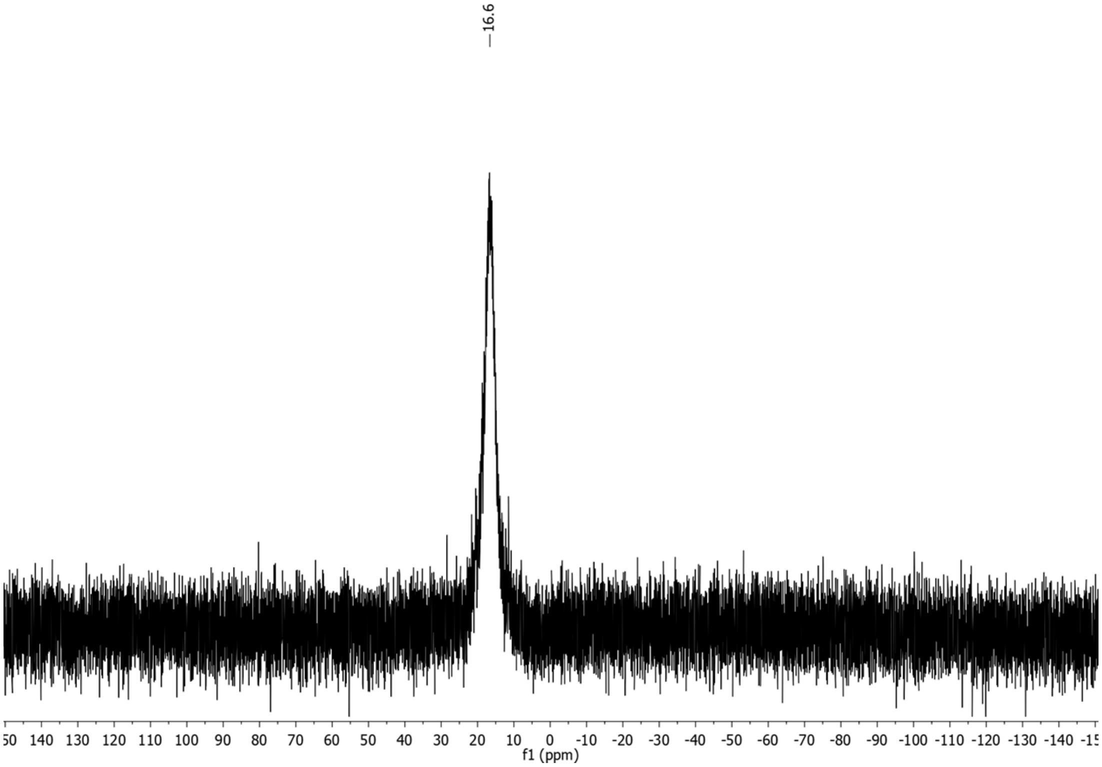

Figure S31. $^{31}P\{^{1}H\}$ NMR spectrum of **3-Sm** recorded from a solution in benzene-d6 at 298 K.

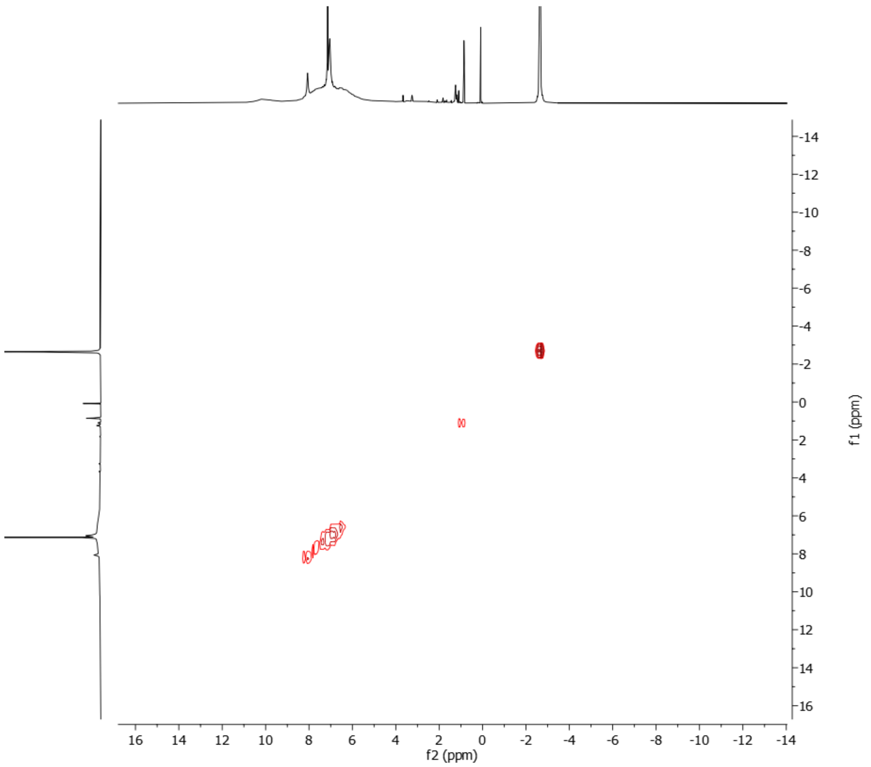

Figure S32. ^1H-^1H COSY NMR spectrum of **3-Sm** recorded from a solution in benzene-d6 at 298 K.

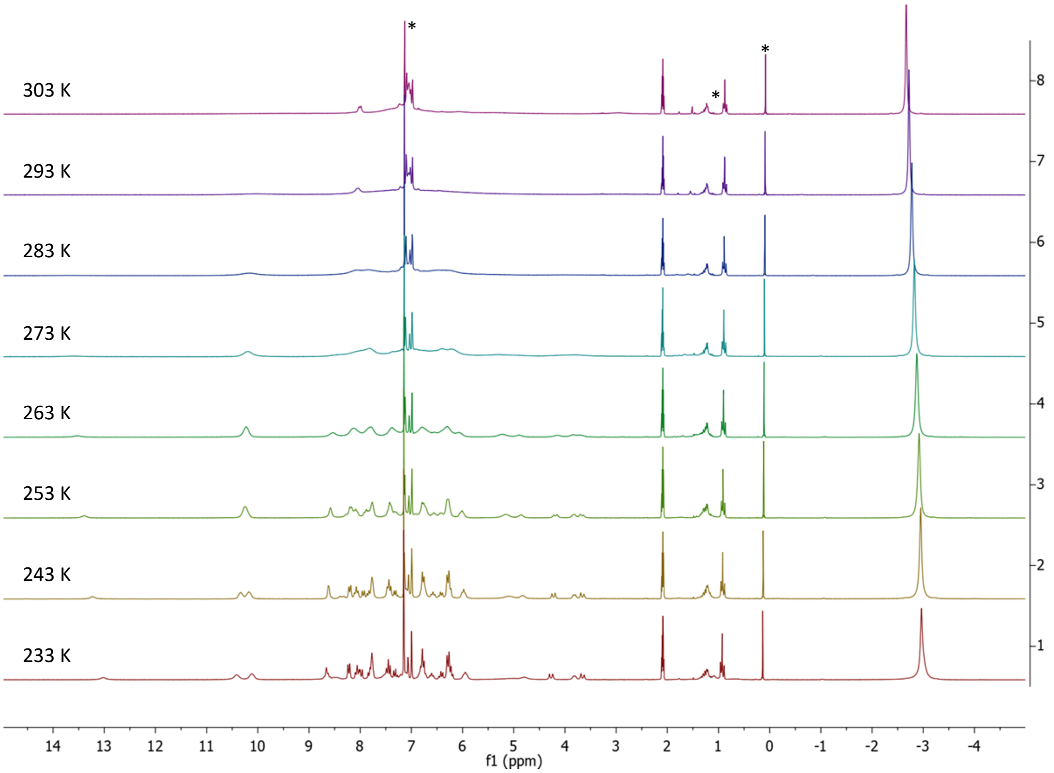

Figure S33. ^1H VT NMR spectra of **3-Sm** recorded from a solution in toluene-d8 from 233 to 303 K in 10 K intervals. Trace impurities are marked with an asterisk.

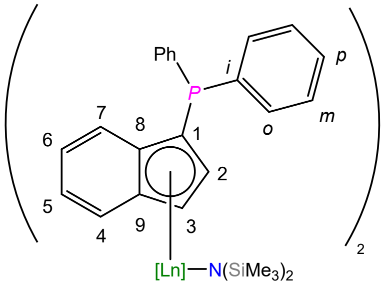

Figure S34: Numbering scheme of the indenyl ligand **1-La** highlighting the individual carbon atoms for assignment via NMR spectroscopy

UV-Vis Spectroscopy:

The UV-Vis absorption spectra are dominated by the absorption of the aromatic systems in the complexes (Figure S35). The phosphinoindenyl complexes **1-La** and **1-Sm** exhibit shoulders at 315 nm ($\varepsilon = 8.9 \cdot 10^3$ $Lmol^{-1}cm^{-1}$) and 335 nm ($\varepsilon = 1.2 \cdot 10^4$ $Lmol^{-1}cm^{-1}$), respectively, which are attributed to the aromatic systems. The samarium derivative exhibits a broad and weak absorption band at 474 nm ($\varepsilon = 5.9 \cdot 10^2$ $Lmol^{-1}cm^{-1}$). The benzonitrile adduct **2** shows a strong absorption shoulder at 316 nm ($\varepsilon = 1.6 \cdot 10^4$ $Lmol^{-1}cm^{-1}$) and a weak broad band at 571 nm ($\varepsilon = 3.7 \cdot 10^2$ $Lmol^{-1}cm^{-1}$). The phosphazidoindenyl complexes **3-La** and **3-Sm** both exhibit shoulders at 317 nm ($\varepsilon = 1.1 \cdot 10^4$ $Lmol^{-1}cm^{-1}$) and 318 nm ($\varepsilon = 1.7 \cdot 10^4$ $Lmol^{-1}cm^{-1}$) as well as weak bands at 571 nm ($\varepsilon = 3.7 \cdot 10^2$ $Lmol^{-1}cm^{-1}$) and 555 nm ($\varepsilon = 1.5 \cdot 10^2$ $Lmol^{-1}cm^{-1}$), respectively.

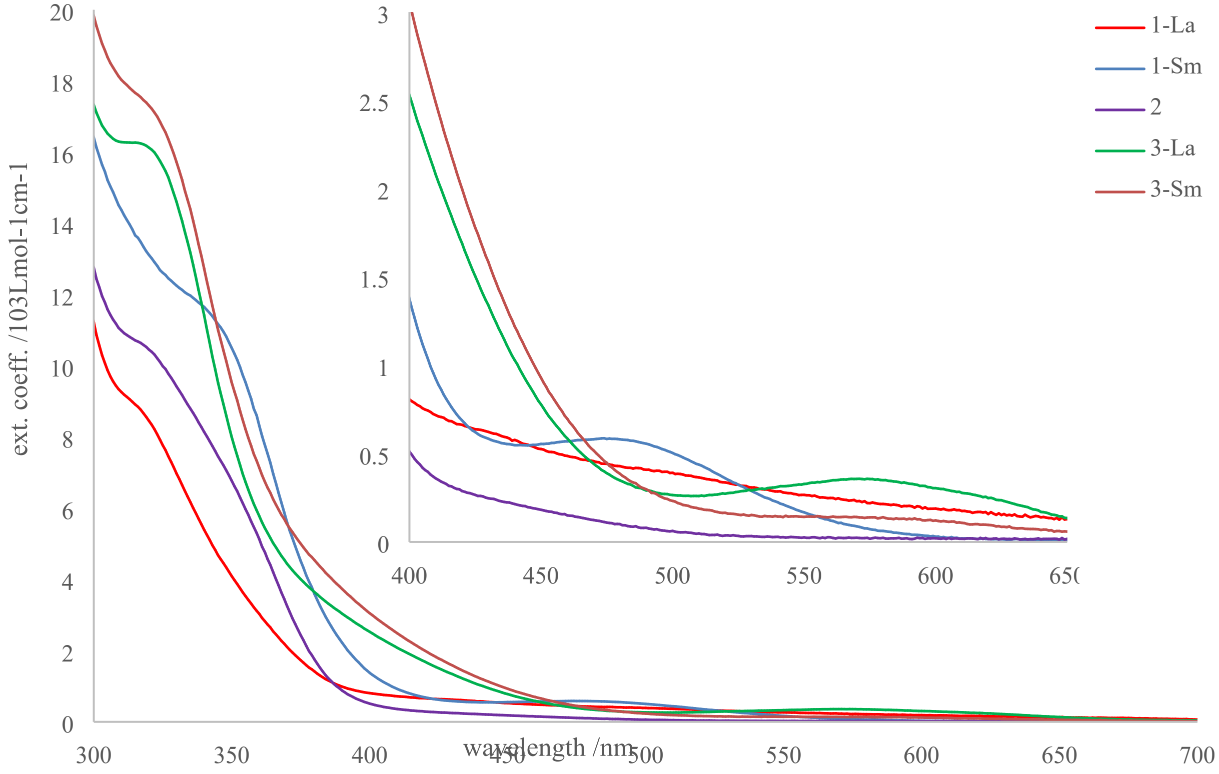

Figure S35. UV-Vis spectra of the complexes **1-La**, **1-Sm**, **2**, **3-La**, and **3-Sm** recorded from 0.05 mM solutions of the compounds in benzene with detail view of range 400 – 650 nm.

Computational Data:

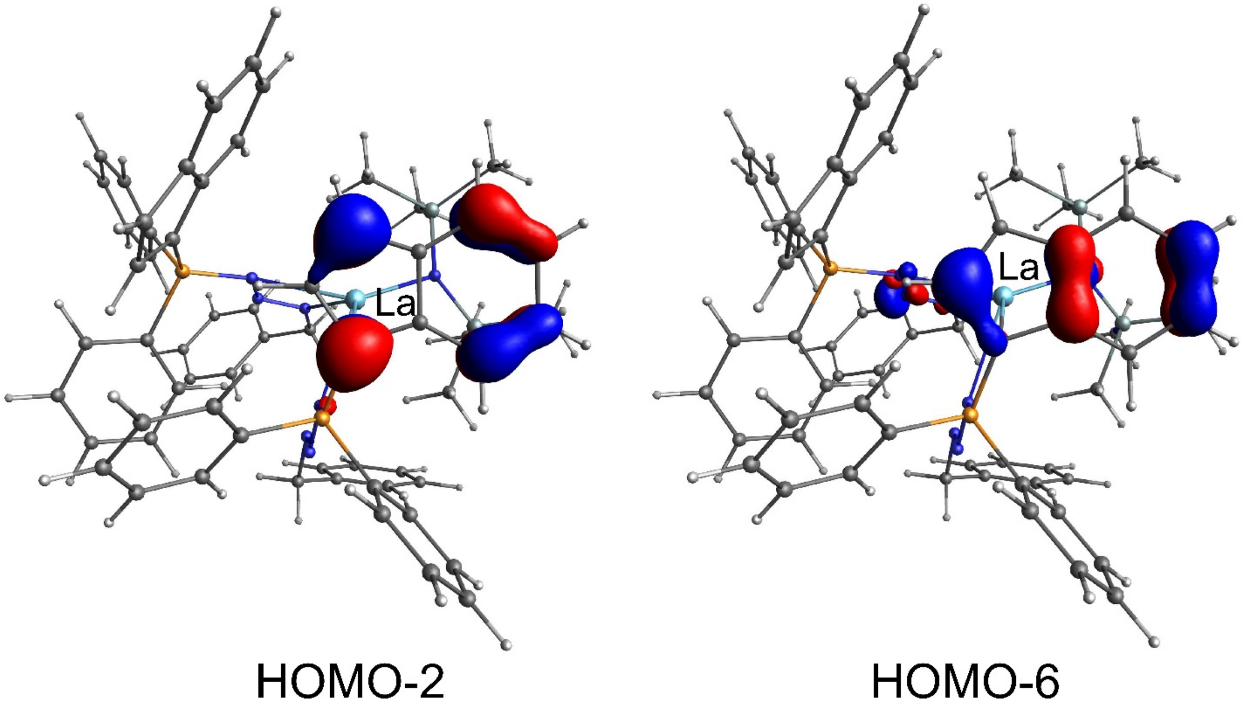

Figure S36. HOMO-2 and HOMO-6 of calculated for **3-La**. DFT calculations performed on the def2-TZVP level of theory using the M06-L functional. The isosurface value for the depicted molecular orbitals is 0.06.

Cartesian coordinates of the optimized structure of **3-La**:

La	6.52638	4.99199	15.84773
P	6.09961	1.96582	13.14114
P	5.61316	7.04396	13.25944
Si	7.82704	4.15809	18.84447
Si	5.76985	6.45022	18.92843
N	4.80234	3.08764	16.38681
N	2.82970	5.37970	15.04389
N	4.94821	2.38020	15.34621
N	6.01933	2.76010	14.61520
N	6.78327	5.31582	18.18651
N	4.74418	6.10075	14.29897
N	3.38118	6.19473	14.26614
C	8.02415	5.66339	13.47703
H	7.80379	4.97189	12.67424
C	9.01383	6.63863	15.28547
C	7.89346	7.46001	14.92448
C	3.63933	3.17481	12.59157
H	3.77630	3.71685	13.51908
C	9.66570	3.12068	11.88379
C	4.76656	7.23280	17.53578
H	5.39306	7.72019	16.78139
H	4.09223	8.00379	17.91329
H	4.12516	6.49942	17.03025
C	9.31819	4.88839	19.70194
H	10.03065	4.12190	20.01085
H	9.02252	5.43536	20.59795

H	9.84288	5.59220	19.05543
C	5.46644	6.53191	11.53763
C	7.25969	6.82822	13.79143
C	9.83202	7.00488	16.36667
H	10.67759	6.38266	16.63738
C	4.20596	6.18612	11.04835
H	3.34714	6.21988	11.70994
C	6.99356	2.96992	20.02800
H	6.67721	3.46643	20.94579
H	7.65912	2.15497	20.31754
H	6.10452	2.52368	19.58022
C	10.69830	2.18257	14.30869
H	11.11623	1.82182	15.24105
C	8.85701	3.49519	10.77186
H	9.20426	3.99122	9.87669
C	7.49486	2.47747	12.30430
C	1.36648	5.52482	15.02989
H	1.08029	6.38037	14.40767
H	0.94219	4.62449	14.57477
C	6.57203	6.49383	10.68739
H	7.55417	6.75187	11.06353
C	2.74453	1.66667	16.64193
C	4.61749	2.28880	12.15407
C	9.34957	2.01303	14.05141
H	8.71114	1.52101	14.77936
C	2.30145	2.67592	10.65597
H	1.39809	2.82139	10.07575
C	5.72243	9.67983	12.44667
H	6.42872	9.31994	11.70607
C	11.53057	2.81251	13.37197
H	12.58428	2.93130	13.59311
C	3.92093	10.60041	14.34658
H	3.21725	10.96000	15.08676
C	9.55546	8.16048	17.05955
H	10.18091	8.45219	17.89409
C	3.61005	2.76271	17.18732
H	3.04556	3.69972	17.26723
H	3.95524	2.52428	18.19658
C	8.82111	2.47960	12.84504
C	4.52202	11.49044	13.46879
H	4.28767	12.54618	13.52315
C	2.48104	3.35945	11.84845
H	1.72258	4.04918	12.20248
C	5.16188	5.74967	8.88809
H	5.04510	5.43505	7.85838
C	8.44249	3.11686	17.38603
H	7.64511	2.56260	16.87225
H	9.13364	2.34735	17.73129
H	9.00655	3.67421	16.62698
C	6.41451	6.10396	9.36596
H	7.27883	6.05979	8.71553
C	7.55842	3.10898	11.02711
H	6.70675	3.26871	10.37912
C	11.02608	3.27594	12.17038
H	11.68325	3.75752	11.45455
C	4.43108	1.60096	10.95262
H	5.18690	0.90255	10.60987

C	6.02364	0.17562	13.42455
C	-0.36493	4.79763	18.33043
H	-0.98621	4.02617	18.76792
C	5.12387	8.78065	13.32991
C	5.42399	11.02967	12.51842
H	5.89413	11.72295	11.83277
C	2.68279	0.41976	17.25190
H	3.25612	0.24011	18.15454
C	3.28355	1.80272	10.20360
H	3.14766	1.26791	9.27187
C	2.00813	1.87383	15.47648
H	2.07635	2.83688	14.98423
C	8.46973	8.97412	16.69601
H	8.28284	9.88675	17.24821
C	4.21938	9.24703	14.28189
H	3.75770	8.55268	14.97238
C	7.10074	-0.62793	13.05479
H	7.98352	-0.16762	12.62576
C	4.87305	-0.41522	13.95522
H	4.02550	0.20024	14.23538
C	4.05751	5.79232	9.72945
H	3.07938	5.51144	9.35964
C	0.06283	4.67551	17.01556
H	-0.21731	3.80531	16.43192
C	0.85812	5.65815	16.43580
C	7.63898	8.63737	15.64971
H	6.81450	9.28742	15.38271
C	4.52324	5.70187	20.11236
H	3.93533	4.91667	19.63381
H	3.82047	6.45551	20.47284
H	4.99707	5.26146	20.98990
C	7.03370	-2.00348	13.22377
H	7.87328	-2.62153	12.93144
C	9.06114	5.52931	14.39005
H	9.82073	4.75958	14.36691
C	6.68896	7.80402	19.83323
H	7.42655	8.27612	19.18439
H	7.22387	7.41044	20.69858
H	6.01656	8.58209	20.19861
C	0.00597	5.90215	19.08260
H	-0.32348	5.99615	20.10954
C	0.79906	6.88946	18.51200
H	1.09063	7.75578	19.09355
C	1.21709	6.76768	17.19757
H	1.83466	7.53866	16.74925
C	5.89198	-2.58431	13.75574
H	5.83826	-3.65908	13.87970
C	4.81335	-1.78913	14.12105
H	3.91709	-2.23813	14.53120
C	1.89785	-0.59474	16.71730
H	1.85682	-1.55892	17.20906
C	1.23105	0.86514	14.93332
C	1.17236	-0.37629	15.55650
H	0.56312	-1.16759	15.13724
H	0.67658	1.04370	14.01942
